# Combating orthopedic implant biofilms — SABER (Study on Agitation for Biofilm Eradication and Reduction) evaluates mechanical, sonication, and radiofrequency approaches: a preclinical in vitro study

**DOI:** 10.2340/17453674.2026.45569

**Published:** 2026-03-31

**Authors:** Timothy G MCMANUS, Michael W FORT, Kaitlyn E BARRACK, Gabrielle S RAY, Michael B SPARKS, Kevin J MCGUIRE, George A O’TOOLE

**Affiliations:** 1 Department of Orthopedics, Dartmouth-Hitchcock Medical Center, Lebanon, NH; 2 Department of Microbiology and Immunology, Geisel School of Medicine at Dartmouth, Hanover, NH, USA

## Abstract

**Background and purpose:**

Medical devices commonly employed in orthopedic surgery continue to be susceptible to challenging and costly biofilm bacterial infections. We aimed to evaluate the impact of mechanical brushing with sonication and radiofrequency on biofilms grown on 3 metallic alloys commonly utilized in orthopedic implants: titanium, stainless steel, and cobalt-chromium.

**Methods:**

Biofilms of 4 common bacteria encountered in orthopedic infections were grown on 540 metal chips for 3 metal alloy cohorts. The biofilms were treated with sterile saline irrigation, sonication brushing, or radiofrequency sonication brushing to compare against untreated control. Biofilm burden was evaluated both qualitatively and quantitatively with scanning electron microscopy imaging and crystal violet (CV) staining optical density or colony-forming unit measurements, respectively. Parametric, nonparametric, and linear regression analyses for quantitative data were performed.

**Results:**

Qualitatively and quantitatively, all interventions showed a strong reduction in biofilm burden of all microbes on all metals. There was a significant decrease in CV-stained biofilms for brushing interventions compared with irrigation alone and controls. Biofilm burden was significantly reduced in all experiments. The untreated control represented 100% biofilm. Irrigation alone reduced biofilm to 44%, while sonication further decreased biofilm to 25%. The most effective method, sonication with radiofrequency, reduced biofilm to 20%.

**Conclusion:**

Our data shows consistent qualitative and quantitative reduction in biofilm burden with brushing interventions compared with irrigation and control. While further study is warranted, our data suggest that mechanical brushing with sonication and radiofrequency may be beneficial tools in reducing biofilm burden on orthopedic metal implants.

Orthopedic implants can harbor infections, leading to devastating complications. Periprosthetic joint infection (PJI) affects 1–3% of hip and knee arthroplasties, causing early failures [1-3]. Such infections present increasing challenges as revision surgeries for PJI are projected to increase by 176% for hips and 170% for knees between 2014 and 2030 [3].

PJI treatment aims for infection eradication and preventing recurrence through medical and surgical interventions. Biofilm formation on implant surfaces complicates treatment, as biofilms protect bacteria within an extracellular polymeric matrix (EPS) [4,5]. These microbial communities are less susceptible to immune defenses and show a 10- to 1,000-fold higher antibiotic tolerance than planktonic bacteria [5,6]. Antibiotic therapy alone is insufficient, and existing surgical strategies warrant improvement to remove these biofilm infections. Biofilm-related infections cause roughly 500,000 deaths annually in the United States [7].

Standard PJI treatment involves surgical irrigation and debridement, implant removal/replacement, and prolonged antimicrobial therapy. Despite this treatment regimen, 2-stage exchange arthroplasty success rates vary between 65% and 95% [8]. This approach also incurs significant physical, mental, and financial burdens, with costs per patient ranging from $30,000–$100,000, overall lifetime costs for total hip arthroplasty PJI of $390,806–$474,004, and total annual costs in the United States projected to reach $1.85 billion by 2030 [9,10].

Research into biofilm prevention and removal is crucial. Preclinical studies suggest promise for biofilm disruption using enzymatic, light-based, sound-based (e.g., ultrasound), or electromagnetic techniques. Sonication, effective against dental biofilms, is used diagnostically in orthopedics to improve culture yields [5,11]. Recently, its potential as a debridement adjunct has gained attention.

We aimed to evaluate mechanical brushing combined with sonication and radiofrequency to reduce biofilm on titanium, stainless steel, and cobalt-chromium alloys. We hypothesized these methods would significantly reduce biofilm burden compared with controls, with sonication and radiofrequency showing the greatest effect.

## Methods

### Microbiology and biofilm assays

Biofilms of *Staphylococcus aureus* Newman, *Staphylococcus epidermidis* ATCC R97-03, *Pseudomonas aeruginosa* PA14, and *Escherichia coli* MG1655 were grown on metal chips made from titanium, stainless steel, or cobalt-chromium for 24 hours. The initial inoculum of each bacterial strain was prepared by growth in 5 mL of tryptic soy broth (TSB) in a test tube on a cell culture roller drum set to a constant speed in a temperature-controlled room at 37° C for ~24 hours. For the biofilm assays, the overnight grown bacterial culture was diluted 1:50 into 30 mL of TSB medium supplemented with 0.2% glucose. Sterile, flat-bottom 12-well polystyrene plates (Corning Cat. #3512) containing the desired metal chip type were then inoculated with 2 mL of bacterial suspension, and the plates incubated at 37° C in a humidified chamber under ambient air (non-CO_2_) conditions for ~24 hours. All treatments followed an identical protocol to ensure consistency for starting inoculum of bacteria. The protocol ensured a 1:50 dilution of the overnight culture (with a bacterial population of ~5 x 10^9^ CFU), thus, the starting inoculum is 1 x 10^8^ in all experiments. For the experiments using the “robust biofilms,” the biofilm was grown for 7 days with fresh medium exchanged every ~24 hours. The biofilms were untreated (control) or treated with either sterile saline irrigation, sonication brushing, or radiofrequency sonication brushing. The extent of biofilm formation was then evaluated by scanning electron microscopy, as reported previously [12,13], or crystal violet staining (CV) and quantification as described below. We also included a direct CFU count to further validate the well-established CV biofilm assay. All bacterial strains and metal alloys were evaluated in triplicate. In total, 540 metal chips were tested (i.e., 108 metal chips per bacterial strain, 36 chips of each metal type per bacterial strain plus the robust biofilm condition) plus some additional chips, as indicated in the text, for control experiments.

### Metal chips as model surfaces

The metal chips were supplied by Xylem Company, which specializes in providing metals to the medical device industry (MN, USA). The metal chips were processed with cleaning solutions (i.e., 70% alcohol spray and dried with Kimtech Science Wipes; Kimberly-Clark Proessional, Roswell, GA, USA) prior to growing the biofilm. After cleaning, the metal chips were placed in sterile 12-well plates until used in the biofilm assays described here.

### Interventions

After biofilm growth on the chips, various treatments were applied for biofilm removal including irrigation with sterile saline, sonication brushing, and radiofrequency sonication brushing for comparison with controls with no intervention. Commercially available electric toothbrushes were used for the brushing interventions: the Acteh JetWave (dual-handle kit with 8 NHB4-model brush heads; 400 Hz; mechanical vibration only, no radiofrequency output; Acteh, Newport Beach, CA, USA), and the Silk’n ToothWave (model TW1PE1001/7290112450821; clinically studied as H7001); 3.3 MHz radiofrequency; 3W max output; Silk’n, Richmond Hill, Ontario, Canada). Only the ToothWave device emits radiofrequency energy, while the JetWave serves as a sonication-only comparator. The interventions were performed by 2 orthopedic surgeons after biofilm growth for 24 hours as described above: each well of the 12-well plate containing the metal chip with the established biofilm was emptied of all medium with a pipette in a laminar flow hood, then the metal chip was placed in a new sterile 12-well plate after the desired treatment was applied. For the “control” group, no interventions were applied apart from gently dipping the metal chip into a phosphate-buffered sterile saline (PBS) wash to rid the metal chip of excess medium and to remove any residual planktonic bacteria. For the “irrigation” intervention arm, 10 mL of phosphate-buffered sterile saline was pipetted over the metal chip (5 mL on each side) prior to placement in a new 12-well plate. For the irrigation arm, 10 mL of phosphate-buffered sterile saline was applied using a powered pipette (Pipet-Aid XP2, Drummond Scientific, Broomall, PA, USA), with 5 mL gently streamed over each side of the metal chip over approximately 5–10 seconds. For the “brushing with sonication” intervention arm, the metal chips were treated for 1 minute (30 seconds each side) with an electric toothbrush prior to placement in the new 12-well plate. The brushing was performed in a consistent manner with the brush bristles coming just to the point of initiating contact with the metal chips with brushing in a circular motion tangential to the surface. Brushing was performed while the chips were immersed in a thin residual layer of PBS to prevent drying and support mechanical and sonication activity. All CFU and CV values were normalized to the surface area (mm²) of the respective metal chip for consistent comparison. Care was taken to not press too firmly such as to contour the bristles or to limit the impact of the sonication. This process was performed identically for the “brushing with radiofrequency sonication” intervention arm. After the mechanical treatments, the chips were gently dipped into PBS wash to remove excess medium and/or any residual planktonic bacteria prior to placement in the new 12-well plate.

### Crystal violet staining and quantifying the biofilms

Crystal violet (CV) staining is an established technique commonly used for quantifying biofilm burden. CV is a base dye that binds to negatively charged bacteria as well as the extracellular matrix components of the biofilm, with a positive relationship between the extent of CV staining and biofilm burden. CV staining and quantification of biofilms has been reported previously [14,15]. Briefly, after the formation of the biofilm and the desired intervention, the metal chips were placed in individual wells of a 12-well plate and covered with 1 mL of CV dye (0.1% w/v in water) for ~10 minutes. The CV was then removed from the wells with a pipette and discarded. The metal chips were then rinsed with 1 mL of water to remove excess CV. The chips were then left to dry in a fume hood at room temperature overnight (~12–24 hours). The CV-stained metal chips were prepared for quantification by applying 1 mL of a destain solution (45% methanol, 10% glacial acetic acid, 45% water) to the wells. The wells were then placed on a rocker table for ~5 minutes to ensure complete solubilization of CV-stained biofilms from the metal chips. 100 *µ*L of solubilized CV solution from each well was then transferred into a well of a 96-well microtiter plate using destain solution as the blank reference well. The absorbance was measured on a plate spectrophotometer (Spectramax M2; Molecular devices LLC, San Jose, CA, USA) at an optical density of 550 nm (OD550). The extent of staining was then normalized to the surface area of the metal chip (mm^2^) before analysis of the data.

### Colony-forming units (CFU) validation and quantifying the biofilms

To complement the quantitative utility of CV analysis, an experiment was performed with *S. epidermidis* on cobalt-chromium in an identical manner to the previous protocol used for CV staining. In this new experiment, after treatments, the remaining biofilm was analyzed by colony-forming units (CFUs) [16] instead of CV staining. The CFUs were enumerated on sheep blood agar plates grown for 16 hours aerobically at 37º C, then normalized to a 1 mL volume and the surface area of the cobalt-chromium metal chips. Output is presented as “Normalized log10(CFU/mL).” Together with the electron microscopy studies, these findings also support and validate the use of the CV assay as a facile tool to measure biofilm formation.

### Statistics

Optical density values from 3 technical replicates per biological replicate from the CV assays were averaged and normalized to surface area (mm^2^) of the respective metal chip. The normalized averages were graphed by treatment, with each biological replicate connected with a line to display trends. Each experiment was performed in triplicate (technical replicates) on 3 different days (biological replicates), yielding 9 data points. The average normalized OD550 of 3 technical replicates of each biological replicate (n = 3) within each treatment group (control, irrigation, sonication, radiofrequency) was plotted and significance indicated on the graph. The mean values of 3 biological replicates were analyzed using 2 different statistical approaches: parametric (ANOVA) and non-parametric (Kruskal–Wallis) using GraphPad Prism (version 10.4.2; https://www.graphpad.com/). In the main figures, one-way ANOVA adjusted for multiple comparisons using Tukey’s test (pairwise) across treatment was used. Supplementary statistical analyses were performed on the same data points described above using Kruskal–Wallis adjusted for multiple comparisons using Dunn’s pairwise multiple comparisons test with a Bonferroni correction.

In our analysis, all fixed effects were categorical, thus the linearity assumption for continuous factors does not apply here. We instead focus on distributional assumptions of the residuals (normality and homoscedasticity), which were assessed using histograms, Shapiro–Wilk tests, Levene’s test, and residual plots. Residual histograms and Shapiro–Wilk tests indicated deviation from normality, so nonparametric Kruskal–Wallis with Dunn’s test (using the Bonferroni correction) was also performed for each dataset. Assumptions for ANOVA include: (i) normal distribution of the data, (ii) homoscedasticity, and (iii) independence of the observations, as defined by the value of 1 observation not influencing the value of another observation. While previous work has suggested that ANOVA is not strictly sensitive to moderate deviations in normal distribution [17-19], we also performed a nonparametric statistical test (Kruskal–Wallis with Dunn’s multiple comparison test with a Bonferroni correction) for each comparison as a supplementary approach. With only 3 biological-replicate days, formal assessment of random-effect normality is not meaningful; however, residual diagnostics did not reveal concerning deviations, and findings were robust across ANOVA, Kruskal–Wallis/Dunn, and mixed-effects models.

Homoscedasticity was tested by plotting a residual plot coupled with a Levene test to address the homogeneity of variance across observations. Both tests suggested homoscedasticity in our dataset. Independence of observations was ensured by the experimental design as each metal chip was used for a single measurement, each microbe was analyzed as a monoculture, and biological replicate (experiment day) was modeled as a random effect to account for any within-day effects. No repeated measurements were performed, thus no such measures were included in the model. Therefore, the categorical variables tested were: metal and treatment. Furthermore, because treatment and metal were orthogonally assigned in our experimental design, multicollinearity among fixed effects was expected to be low. This assumption was confirmed by examining the correlation matrix of the fixed-effect estimates (all absolute correlations < 0.7), indicating no meaningful multicollinearity.

To address significant changes in biofilm burden across all variables in the dataset (i.e., microbe, metal, treatment, biological replicate), a mixed effect linear model from the R package lme4 was used [20]. In this analysis, all 9 datapoints were included in the dataset and were assigned with appropriate metadata (Table S1, see Supplementary data). Biological replicate was set as the random variable to control for variation between experiment days. Because each microbe was grown individually in each experiment, we evaluated statistical significance by subsetting all data from each microbe, then running a mixed effect linear model on each microbe, resulting in 4 different models. Fixed variables included: Treatment + Metal + Treatment*Metal. We chose this statistical approach because it allows for inclusion of all controlled and random variables in the dataset. Multiple comparisons were adjusted for using the Bonferroni correction. All the 95% confidence intervals (CI) for our analyses are included in Supplementary Tables 2–35.

Assumptions for a linear model include: (i) normal distribution of the data, (ii) homoscedasticity, and (iii) independence of the observations. We include marginal and conditional R2 values in Supplementary Tables 26–29 to summarize the proportion of variance explained by the fixed and random effects.

### Selection of bacterial strains and metals

The 4 bacterial strains tested in this study—*S. aureus* Newman, *S. epidermidis* ATCC R97-03, *P. aeruginosa* PA14, and *E. coli* MG1655—represent some of the most implicated Gram-positive and Gram-negative organisms in implant-related infections in orthopedics [6,21]. The strains used here were selected because previous studies showed robust biofilm formation [15,22-25].

The metals selected included 3 metal alloys commonly utilized in orthopedic implants: titanium (ASTM F67, Ti GR-1), cobalt-chromium (MP35N, ASTM F688), and stainless steel (316L SS, ASTM A240), which represents a closely related alloy within the same compositional class. [26]. The metal chips were consistent in their size and nature (i.e., diameter, thickness, surface roughness, etc.). The average size of the chips is as follows: titanium (64 mm^2^), stainless steel (66 mm^2^), and cobalt-chromium (109 mm^2^). All chips were ~0.5 mm thick.

### Funding, use of AI tools, and disclosures

This work was supported by funding from the Elmer R. Pfefferkorn, PhD chair to GAO. Additional support from the NIH (T32HL134598) was granted to KEB.

AI tools were not used in this study.

All authors certify no conflicts of interest, funding, or commercial associations (e.g., consultancies, equity, patents) relevant to this work, including immediate family members. TGMc filed intellectual property for a potential surgical infection treatment device, with no industry involvement or monetary gains. Complete disclosure of interest forms according to ICMJE are available on the article page, doi: 10.2340/17453674.2026.45569

## Results

### Interventions with S. aureus biofilms

**Figure 1:**
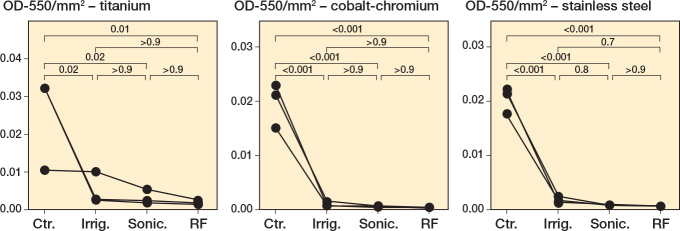
*Staphylococcus aureus* biofilms decrease following all treatments. *S. aureus* Newman was grown for 24 hours under static conditions on titanium, cobalt-chromium, and stainless steel. Following 24 hours’ incubation, the biomass was left untreated (Ctr.), irrigated with PBS (Irrig.), or treated by brushing with sonication (Sonic.), or brushing with sonication with radiofrequency (RF). The remaining biofilm was stained with CV, quantified, and normalized to the area (mm^2^) of the respective metal. 3 biological replicates for each treatment group (performed in triplicate) are plotted, with lines connecting each biological replicate across all treatments. Statistical analyses of normalized biofilms were performed using one-way ANOVA with Tukey’s multiple comparisons.

We first established *S. aureus* biofilms on metal chips, subjected the biofilms to each intervention, then examined the remaining biofilm biomass using crystal violet (CV) staining. Qualitatively and quantitatively, all interventions showed a strong reduction in biofilm burden of *S. aureus* on all metals (Figure 1, Tables S2–S4, Figure S1). As a control, we showed that the metal chips, prior to the start of the experiment, had no detectable microbial contamination (Figure S2). As a supplementary approach, we analyzed these results using a nonparametric statistical test, Kruskal–Wallis with Dunn’s multiple comparisons test with a Bonferroni correction, to account for variations in normal distribution. Results from this analysis support the significant reduction in biofilm following treatment with radiofrequency brushing on all metals (Figure S3, Tables S5–S7).

**Figure 2:**
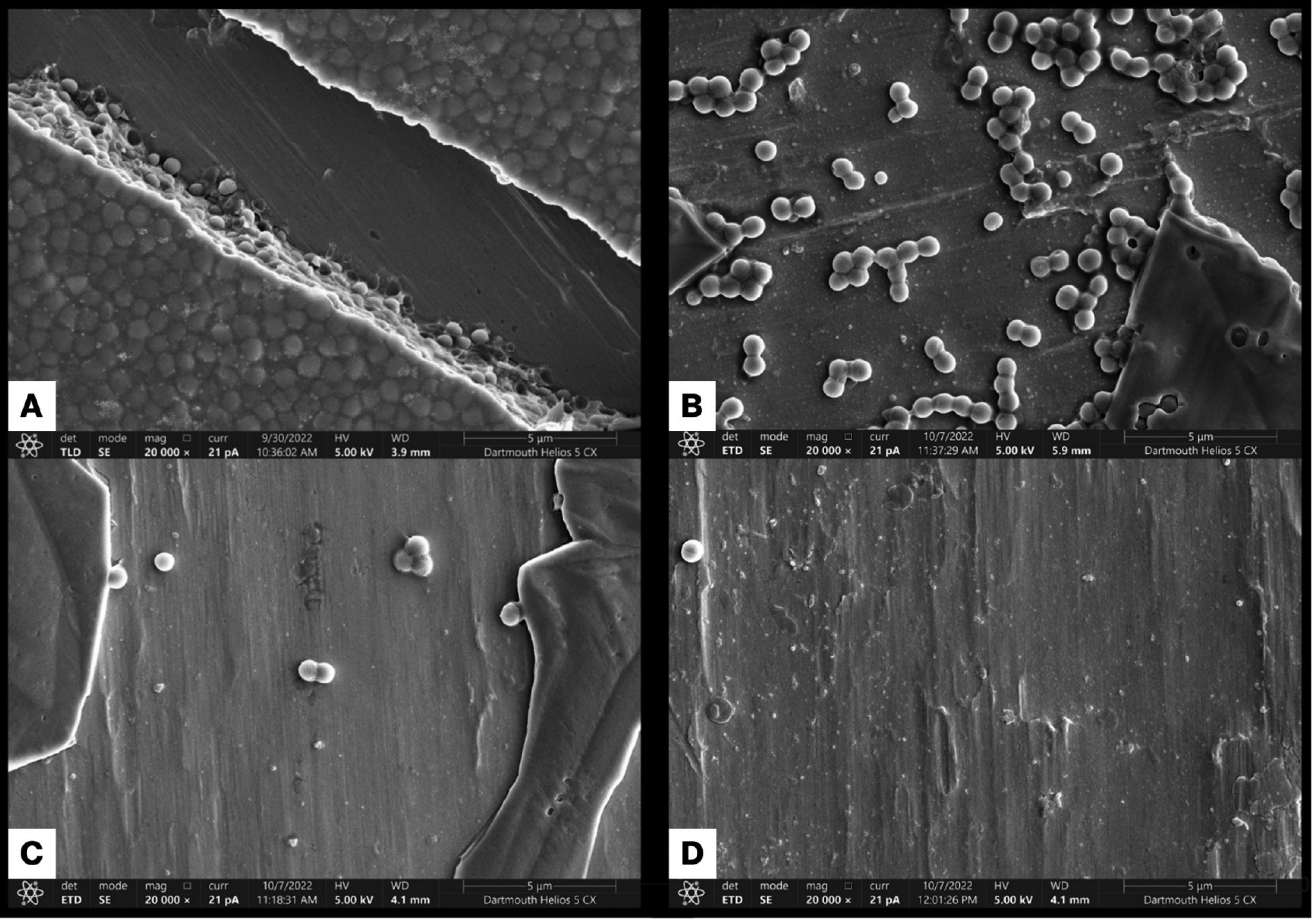
Scanning electron microscopy. Scanning electron microscopy of *S. aureus* Newman biofilms grown for 24 hours under static conditions on cobalt-chromium and treated as follows: (A) untreated control, (B) irrigation with sterile saline, (C) brushing with sonication, or (D) brushing with sonication with radiofrequency. The samples were prepared as described in the Methods.

To complement the CV assays, we used scanning electron microscopy (SEM). To perform the SEM studies, we first established *S. aureus* biofilms on metal chips, subjected the biofilms to each intervention, then examined the remaining biofilm biomass using SEM by randomly selecting fields of view. SEM shows a robust biofilm for the untreated control (Figure 2A), a notable reduction in the adhered bacteria for the irrigation treatment (Figure 2B) and very few adhered bacterial cells for either brushing treatment (Figure 2C–D). Additional images are shown in the supplementary data confirming this finding (Figure S4). While we can only show a small portion of the surface of each chip using this approach, the random selection of the fields of view confirms the substantial removal of the biofilm after brushing treatments.

### Interventions with biofilms formed by other microbes of relevance to PJI

**Figure 3:**
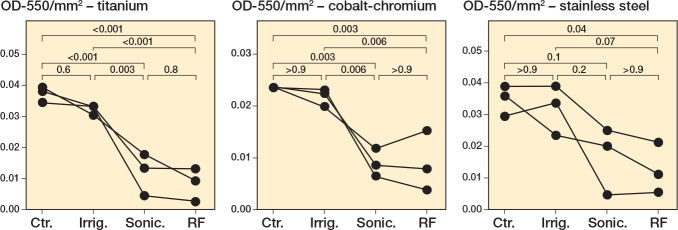
*Staphylococcus epidermidis* biofilms decrease following sonication and radiofrequency. *S. epidermidis* ATCC R97-03 was grown for 24 hours under static conditions on titanium, cobalt-chromium, and stainless steel. Following 24 hours’ incubation, the biomass was left untreated (Ctr.), irrigated with PBS (Irrig.), or treated by brushing with sonication (Sonic.), or brushing with sonication with radiofrequency (RF). For details on staining and statistics, see Figure 1.

As was observed for *S. aureus*, qualitatively and quantitatively, all interventions showed a strong reduction in biofilm burden for *S. epidermidis*. For the *S. epidermidis* biofilms, there was a significant decrease in CV staining of the biofilm for the brushing interventions compared with control (sonication: P < 0.001, radiofrequency: P < 0.001; Figure 3, Table S8) or irrigation (sonication: P = 0.003, radiofrequency: P < 0.001; Table S8) on titanium. Similar observations were consistent on cobalt-chromium-grown biofilms (Figure 3, Figure S5, Table S9). Only brushing with radiofrequency sonication resulted in a significant reduction of the biofilm for the *S. epidermidis* biofilms on stainless steel (P = 0.04; Figure 3, Table S10). Of note was the lack of efficacy of irrigation for removing the biofilm of *S. epidermidis* (Figure 3, Figure S5). Again, we analyzed these results using a nonparametric statistical test, Kruskal–Wallis with Dunn’s multiple comparisons test with a Bonferroni correction, to account for variations in normal distribution. Results from this analysis support the significant reduction in biofilm following treatment with sonication and radiofrequency brushing across metals (Figure S6, Tables S11–S13).

As an additional control, we quantified the 24-hour *S. epidermidis* biofilms using direct counting by colony forming units (Figure S7), which showed the same pattern as CV staining for each of the treatments. That is, only the mechanical treatments showed a significance reduction in bacterial burden compared with the control.

**Figure 4:**
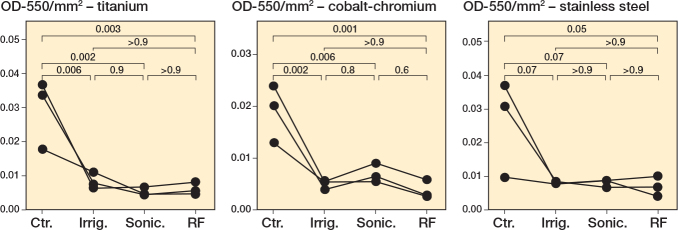
*Pseudomonas aeruginosa* biofilms decrease following treatments in a metal-dependent manner. *P. aeruginosa* PA14 was grown for 24 hours under static conditions on titanium, cobalt-chromium, and stainless steel. Following 24 hours’ incubation, the biomass was left untreated (Ctr.), irrigated with PBS (Irrig.), or treated by brushing with sonication (Sonic.), or brushing with sonication with radiofrequency (RF). For details on staining and statistics, see Figure 1.

**Figure 5:**
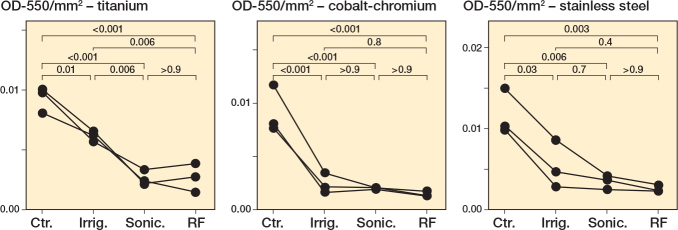
*Escherichia coli* biofilms decrease following all treatments. *E. coli* MG1655 was grown for 24 hours under static conditions on (A) titanium, (B) cobalt-chromium and (C) stainless steel. Following 24 hours’ incubation, the biomass was left untreated (Ctr.), irrigated with PBS (Irrig.), or treated by brushing with sonication (Sonic.), or brushing with sonication with radiofrequency (RF). For details on staining and statistics, see Figure 1.

Similar to what was observed for *Staphylococcus* spp., there was a significant decrease in CV-stained *P. aeruginosa* (Figure 4, Figure S8, Tables S14–S19) and *E. coli* (Figure 5, Figure S9, Tables S20–S25) biofilms for brushing interventions compared with irrigation and controls.

Overall, interventions demonstrated significant decrease (as determined by one-way ANOVA) in CV-stained biofilm burden compared with control for all bacteria and metals with the following exceptions: *S. epidermidis* control compared with irrigation for all metal chips; *S. epidermidis* control compared with sonication brushing on stainless steel; and *P. aeruginosa* control compared with all interventions on stainless steel.

### Analysis as a function of metal types

Next, we tested the role of surface type on biofilm. To do so, we used a mixed effect linear model to account for all variables in the dataset, including metal type, the microbe grown (*S. aureus, S. epidermidis, P. aeruginosa, E. coli*) and the treatment performed on the biofilm. As each microbe was grown independently, we subset the data by microbe and ran 4 individual mixed effect models. Results of the mixed effect models performed on each microbe suggest that sonication brushing and radiofrequency sonication brushing reduced CV-stained biofilm after adjusting for multiple comparisons (Tables S26–S29). Furthermore, *S. aureus* (P adj. < 0.001) and *P. aeruginosa* (P adj. = 0.001) biofilms were reduced following irrigation alone in a metal-independent manner (Tables S26 and S28, respectively). Notably, biofilm formation and response to treatment were largely unchanged across metal types in all microbes after multiple comparisons, although, before adjusting for multiple comparisons, cobalt-chromium showed reduced quantities of *S. epidermidis* (P = 0.006) and *P. aeruginosa* (P = 0.03) biofilms (Tables S27–S28). We did not observe significant interactions between treatment and metal variables, supporting that the effect of treatment does not depend on metal type, and vice versa.

Additionally, the use of the mechanical brushing with sonication and/or radiofrequency reduced CV-stained biofilm qualitatively and quantitatively for all 3 metals when compared with irrigation alone as well as the untreated control. We found that, across all experiments, the average magnitude of biofilm reduction increased with sonication (the biofilm remaining was 25% of untreated control) and sonication plus radiofrequency (20% of untreated control) when compared with irrigation alone (44% of untreated control) (Figure S10). Of note, *S. aureus* biofilms were most susceptible to treatment and *S. epidermidis* biofilms were least susceptible (Figure S10). Thus, sonication and sonication plus radiofrequency reduced the biofilm burden beyond standard irrigation in the tested conditions.

### Interventions with robust S. aureus biofilms

**Figure 6:**
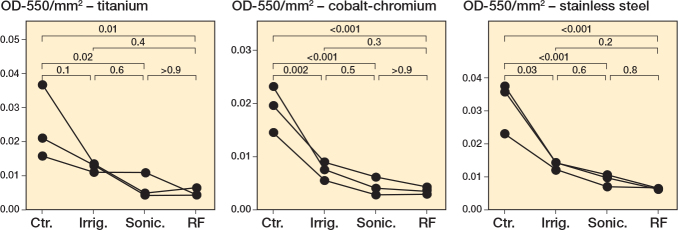
*Staphylococcus aureus* robust biofilms decrease following treatments in a metal-dependent manner. *S. aureus* Newman was grown for 7 days under static conditions, with fresh medium added every 24 hours, on titanium, cobalt-chromium, and stainless steel. Following 24 hours’ incubation, the biomass was left untreated (Ctr.), irrigated with PBS (Irrig.), or treated by brushing with sonication (Sonic.), or brushing with sonication with radiofrequency (RF). For details on staining and statistics, see Figure 1.

To further extend our analysis, we tested each treatment intervention vs a “robust” biofilm of *S. aureus* that was grown for 7 days, rather than the typical 24 hours. Compared with the untreated control, both brushing interventions resulted in a significant reduction in the biofilm burden on all metals (Figure 6, Tables S30–S32). Irrigation resulted in a significant reduction in the biofilm for cobalt-chromium (P = 0.002, Table S31) and stainless steel (P = 0.003, Table S32), but not for titanium (P = 0.1, Table S30). As was observed for the experiments in Figures 1–5, sonication plus radiofrequency resulted in the largest reduction in biofilm biomass (Figure 6). Similarly, we performed supplementary nonparametric statistical tests to account for variances in distribution (Figure S11, Tables S33–S35). These results support the negative effect of sonication (P < 0.001 for titanium, P < 0.001 for cobalt-chromium, P < 0.001 for stainless steel) and radiofrequency (P < 0.001 for all metals) treatment on robust biofilms grown on all metals tested, compared with control robust biofilms (Figure S11A–C, Tables S33–S35).

## Discussion

We investigated the impact of irrigation, sonication brushing, and sonication plus radiofrequency brushing on biofilms of 4 microbes relevant to PJI across 3 orthopedic implant metals [26]. The biofilm extent varied among microbes and metals, reflecting the complexity of treating such infections. We showed that all treatments decreased biofilm biomass irrespective of metal type. This conclusion was based both on significance testing using multiple tests, as well as analysis of the 95% confidence intervals, which supported the statistical tests. Irrigation, a standard PJI treatment, reduced biofilm across most conditions, but its limitations are concerning. *S. epidermidis*, for example, showed minimal reduction. A prior study found that irrigation alone reduced biofilm by less than tenfold, insufficient to prevent recurrence [27]. This data aligns with reported failure rates of 30–50% for debridement, antibiotics, and implant retention (DAIR) in PJI [28]. This finding emphasizes the value of analyzing the different treatment effects across microbes and metal types.

Sonication offers an adjunctive strategy against biofilms. We observed greater biofilm reduction with sonication and sonication plus radiofrequency compared with irrigation alone. Sonication, widely used in dentistry and diagnostics, disrupts biofilms through cavitation and microstreaming, improving culture yield and bacterial removal [11,29-33]. Prior studies have confirmed its efficacy in removing biofilms from orthopedic implant surfaces [33-36]. Our findings align with these results and expand them to cobalt-chromium surfaces and multiple microbes, including Gram-positive and Gram-negative organisms, in addition to evaluating sonication with radiofrequency treatment.

Our study used toothbrush-based devices operating at sub-ultrasonic frequencies (400 Hz, 3.3 MHz), which differ from standard diagnostic sonicators but demonstrated effective biofilm reduction. While not yet suited for intraoperative use, the concept may have treatment or diagnostic potential in orthopedics. Further engineering and development will be needed to translate this into a surgical instrument optimized for clinical application.

This study’s limitations include its preclinical design with inability to artificially replicate host factors or polymicrobial infections [29,37]. Testing multiple clinical isolates and continued efforts to minimize human error should be explored in future studies.

### Conclusions

Irrigation, sonication brushing, and sonication plus radiofrequency brushing significantly reduced biofilm on 3 common orthopedic metals in vitro. Sonication and radiofrequency brushing demonstrated superior reductions compared with irrigation alone. While further study is warranted, these methods show promise as tools to reduce biofilm burden on orthopedic implants.

From a clinical perspective, although implant exchange remains standard for many implant infections and PJI, improving the success of DAIR procedures remains a critical goal. Enhanced mechanical biofilm disruption beyond irrigation alone may augment intraoperative debridement, potentially improving DAIR success rates and ultimately increasing the feasibility of durable implant retention.

### Supplementary data

Supplementary Figures 1–11 and Supplementary Tables 1–35 are available as 3 Supplementary data files on the article home page: doi: 10.2340/17453674.2026.45569

## Supplementary Material







## Notes

Contributions are consistent with the 4 criteria of ICMJE. Design of study: GAO, TGMc, MWF, MBS, KJMc. Data acquisition: TGMc, MWF, GAO, KEB. Data analysis: GAO, KEB, TGMc, MWF. Data interpretation: GAO, KEB, TGMc, MWF. Paper draft: TGMc, GAO, GSR. Critical review: all authors.

The authors would like to thank the staff at the Electron Microscope Facility at Dartmouth for their assistance in producing wonderful SEM images of biofilm for our study.

Handling co-editors: Marianne Westberg and Philippe Wagner

*Acta* thanks Geir Bjerkan and Tor Johan Monsen for help with peer review of this manuscript.
