# Supplementary material for: Combating orthopedic implant biofilms — SABER (Study on Agitation for Biofilm Eradication and Reduction) evaluates mechanical, sonication, and radiofrequency approaches: a preclinical in vitro study
Source: Acta Orthop. 2026 Mar 31;97:209–16. doi: 10.2340/17453674.2026.45569 (PMC13037461; doi:10.2340/17453674.2026.45569)
Supplement: Supplementary file 1 [file ActaO-97-45569-s1.pdf]

Supplemental Figures

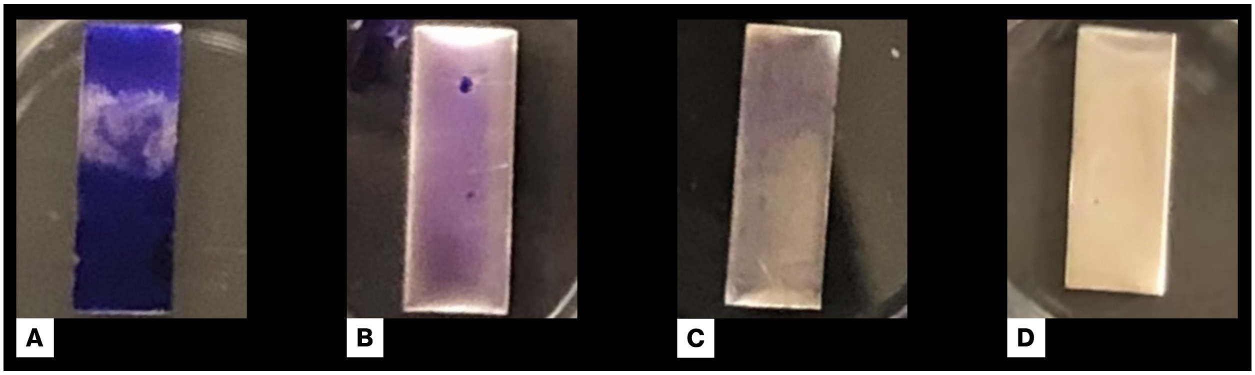

Figure S1. Crystal violet (CV) staining of *S. aureus* biofilms. Shown is CV staining of *S. aureus* Newman biofilms formed on stainless steel for 24 hours then treated as follows: (A) Untreated control, (B) irrigation with sterile saline, (C) brushing with sonication, (D) brushing with sonication and radiofrequency.

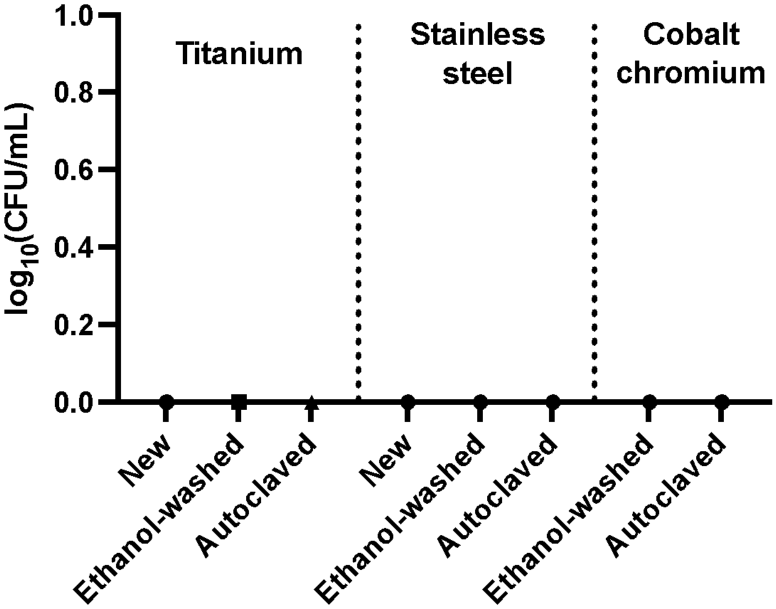

Figure S2. New and sterilized metals do not have microbial contamination. Each metal was tested for contamination at various stages: "new" or fresh from purchased, ethanol-washed and following autoclaving. Autoclaving served as a control because no viable bacteria would be expected to be detected post-autoclave treatment. The metals were placed in sterile PBS, then PBS was plated on sheep blood agar in serial dilutions and grown aerobically for 24h. No growth was observed for any treatment.

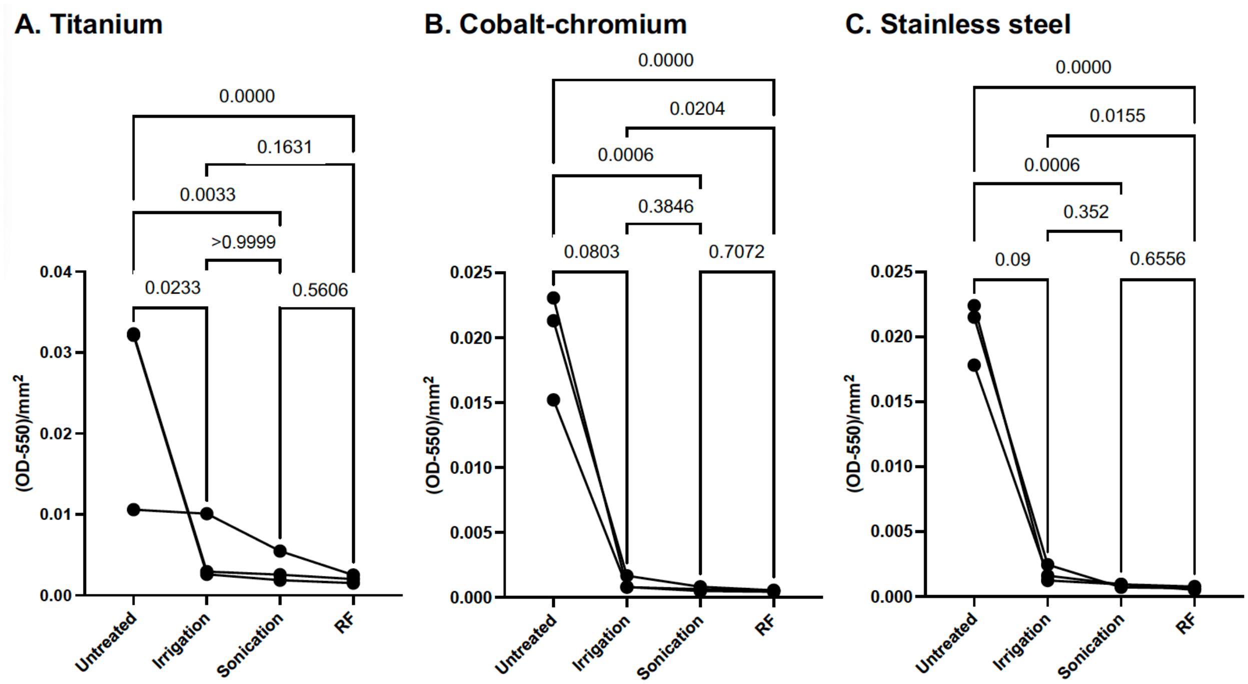

Figure S3. ***Staphylococcus aureus* biofilms decrease following treatment with sonication and radiofrequency.** *S. aureus* Newman was grown for 24 h under static conditions on (A) titanium, (B) cobalt-chromium and (C) stainless steel. Following 24 h incubation, the biomass was left untreated (Control), irrigated with PBS (Irrigation), or treated by brushing with sonication (Sonication) or brushing with sonication with radiofrequency (RF). The remaining biofilm was stained with CV, quantified and normalized to the area (mm^2) of the respective metal. Three biological replicates for each treatment group (performed in triplicate) are plotted, with lines connecting each biological replicate across all treatments. Statistical analyses of normalized biofilms were performed using Kruskal-Wallis with Dunn's multiple comparisons with a Bonferroni correction.

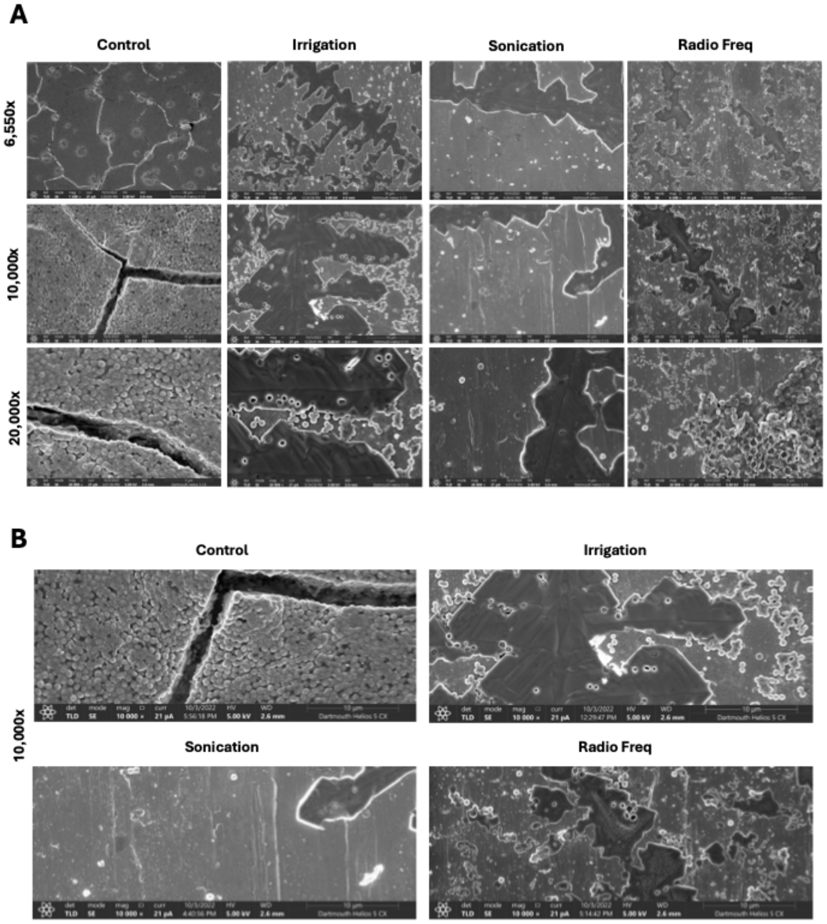

Figure S4. Additional SEM images. A. Scanning electron microscopy of *S. aureus* Newman biofilms grown for 24h under static conditions on cobalt-chromium and treated as follows: (A) untreated control, (B) irrigation with sterile saline, (C) brushing with sonication, or (D) brushing with sonication with radiofrequency. Shown are selected images at 6,500x, 10,000x and 20,000x magnifications. The samples were prepared as described in the Materials and Methods. **B**. Zoomed in view of a portion of the 10,000x magnifications shown in panel A.

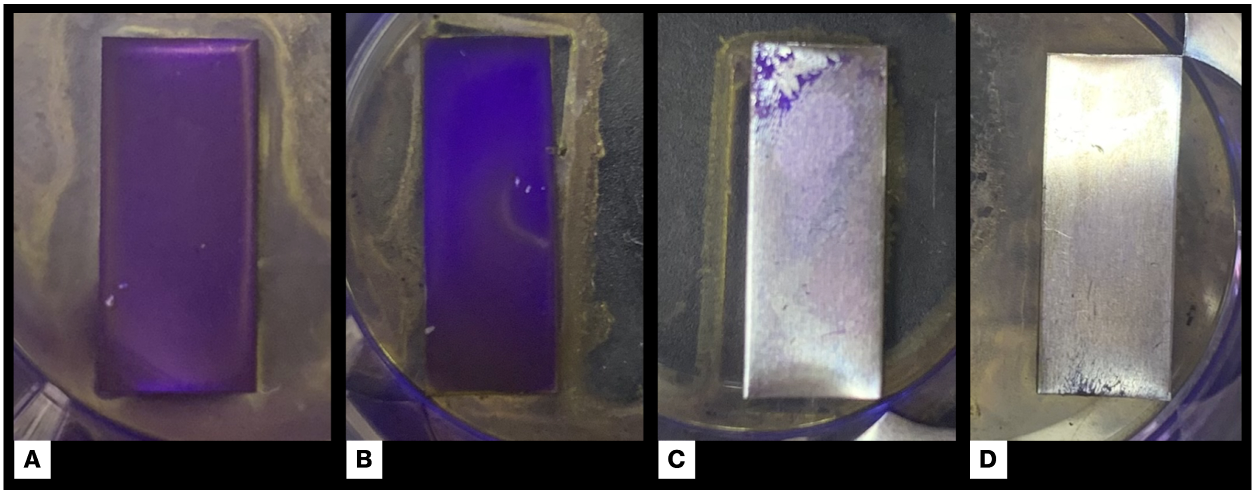

Figure S5. Crystal violet (CV) staining of *S. epidermidis* biofilms. Shown is CV staining of *S. epidermidis* ATCC R97-03 biofilms formed on stainless steel for 24 hours then treated as follows: (A) Untreated control, (B) irrigation with sterile saline, (C) brushing with sonication, (D) brushing with sonication and radiofrequency.

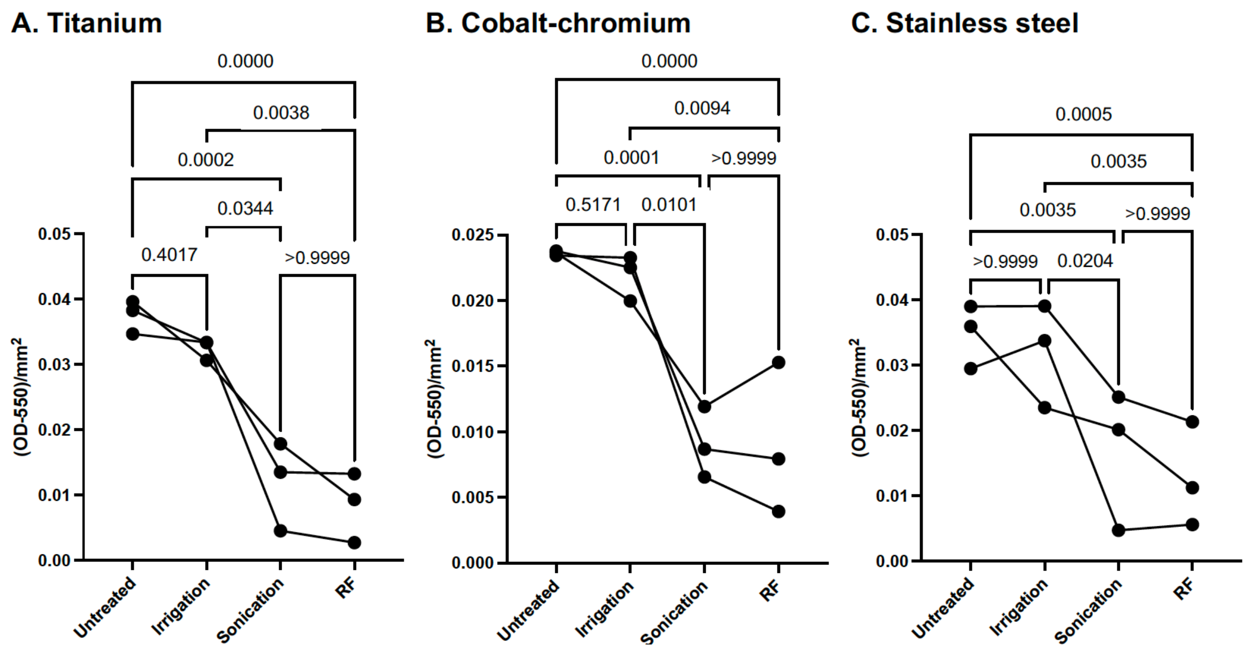

Figure S6. *Staphylococcus epidermidis* biofilms decrease following sonication and radiofrequency across all metals. *S. epidermidis* ATCC R97-03 was grown for 24 h under static conditions on (A) titanium, (B) cobalt-chromium and (C) stainless steel. Following 24 h incubation, the biomass was left untreated (Control), irrigated with PBS (Irrigation), or treated by brushing with sonication (Sonication) or brushing with sonication with radiofrequency (RF). The remaining biofilm was stained with CV, quantified and normalized to the area (mm^2) of the respective metal. Three biological replicates for each treatment group (performed in triplicate) are plotted, with lines connecting each biological replicate across all treatments. Statistical analyses of normalized biofilms were performed using Kruskal-Wallis with Dunn's multiple comparisons with a Bonferroni correction.

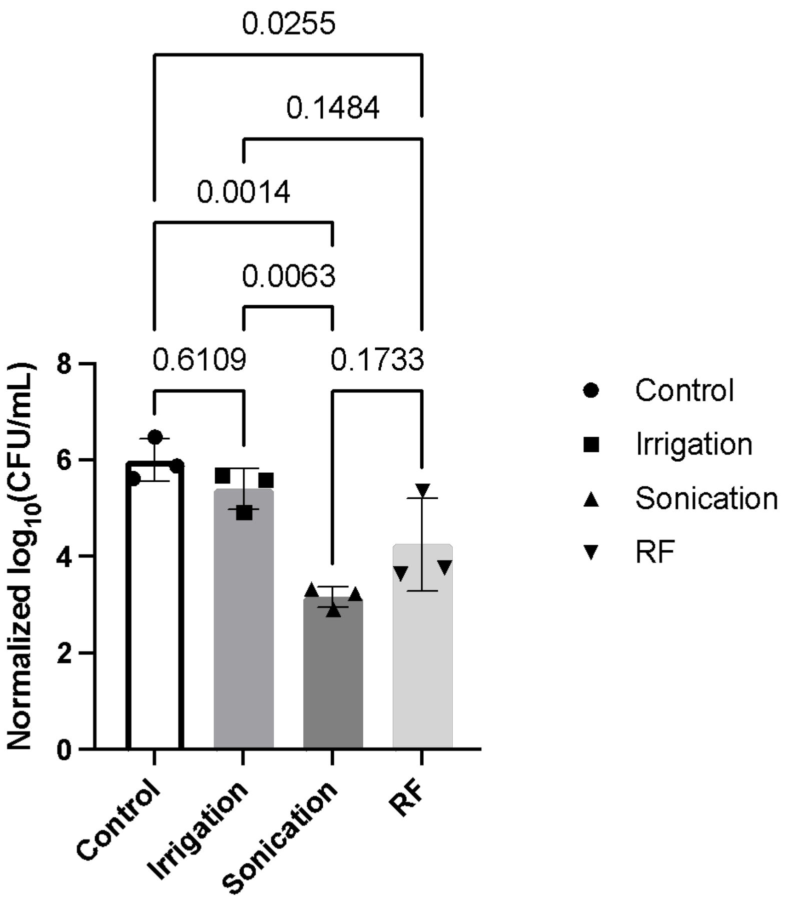

Figure S7. Mechanical treatments reduce *Staphylococcus epidermidis* biofilm burden as assessed by CFUs at 24h. Colony forming units per milliliter (CFU/mL) are quantified and normalized by 1 mL volume and surface area (mm^2) following each treatment, relative to untreated (control) for *S. epidermidis* grown on cobalt-chromium for 24h. On cobalt-chromium, sonication significantly reduces the *S. epidermidis* biofilm burden relative to control and irrigation, while radiofrequency (RF) significantly reduces the *S. epidermidis* biofilm burden relative to control. Statistical analysis performed using one-way ANOVA with Tukey's multiple comparisons.

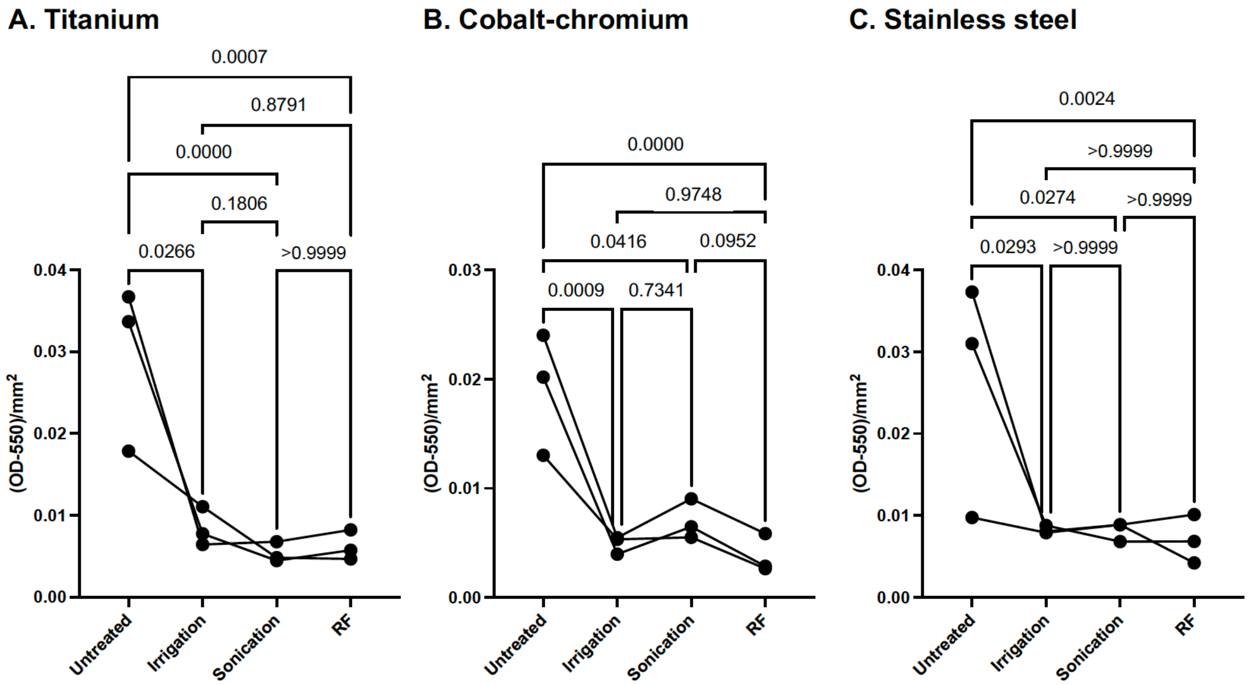

Figure S8. ***Pseudomonas aeruginosa*** **biofilms significantly decrease following treatments across metals.** *P. aeruginosa* PA14 was grown for 24 h under static conditions on (**A**) titanium, (**B**) cobalt-chromium and (**C**) stainless steel. Following 24 h incubation, the biomass was left untreated (Control), irrigated with PBS (Irrigation), or treated by brushing with sonication (Sonication) or brushing with sonication with radiofrequency (RF). The remaining biofilm was stained with CV, quantified and normalized to the area (mm^2) of the respective metal. Three biological replicates for each treatment group (performed in triplicate) are plotted, with lines connecting each biological replicate across all treatments. Statistical analyses of normalized biofilms were performed using Kruskal-Wallis with Dunn's multiple comparisons with a Bonferroni correction.

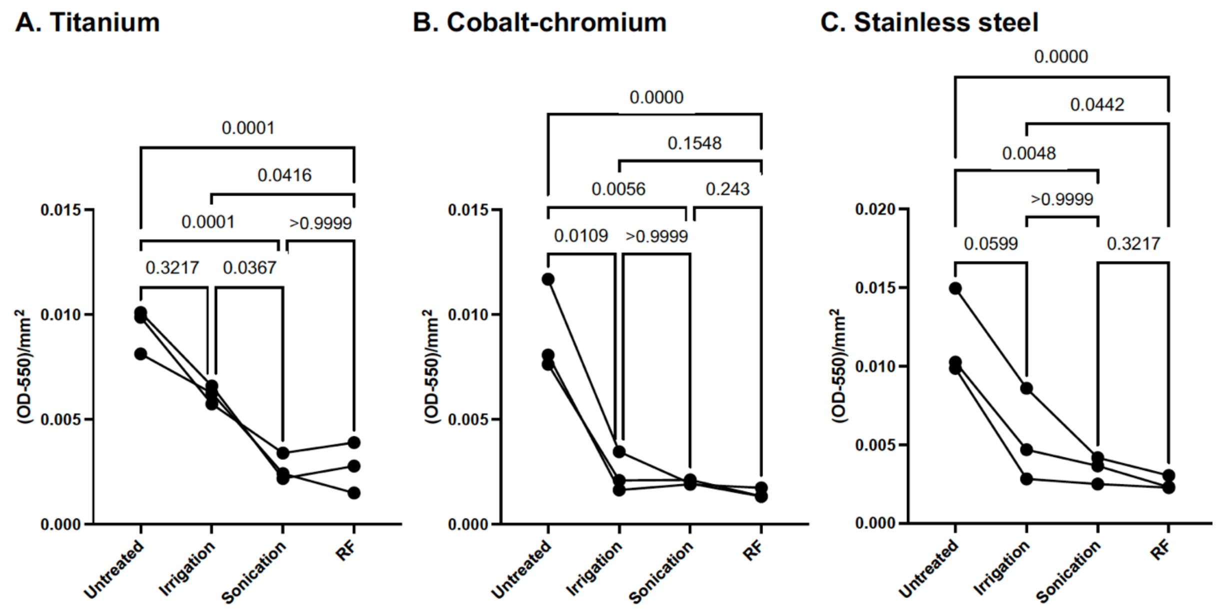

Figure S9. *Escherichia coli* biofilms decrease following sonication and radiofrequency treatment in a metal-independent manner. *E. coli* MG1655 was grown for 24 h under static conditions on (**A**) titanium, (**B**) cobalt-chromium and (**C**) stainless steel. Following 24 h incubation, the biomass was left untreated (Control), irrigated with PBS (Irrigation), or treated by brushing with sonication (Sonication) or brushing with sonication with radiofrequency (RF). The remaining biofilm was stained with CV, quantified and normalized to the area (mm^2) of the respective metal. Three biological replicates for each treatment group (performed in triplicate) are plotted, with lines connecting each biological replicate across all treatments. Statistical analyses of normalized biofilms were performed using Kruskal-Wallis with Dunn’s multiple comparisons with a Bonferroni correction.

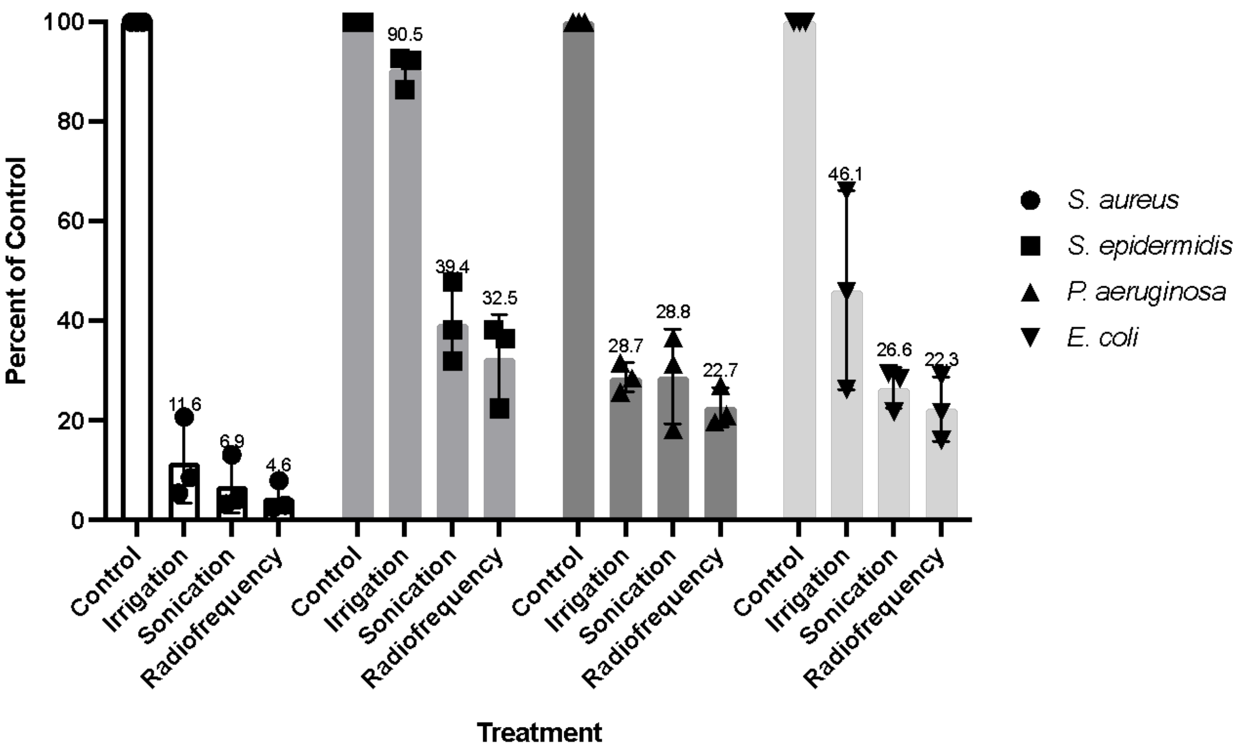

Figure S10. Percent CV-stained biofilm relative to untreated control for all microbes across all metals. Percent of control (Y-axis) is plotted across each treatment group (X-axis) for *S. aureus* (circle), *S. epidermidis* (square), *P. aeruginosa* (upright triangle), *E. coli* (inverted triangle). Untreated control values are normalized to 100% and the average normalized OD_{550} value for each microbe on each metal is plotted relative to the untreated control biofilm. Each point on the plot depicts the average value (of 9 datapoints) per metal, thus there are three points (titanium, cobalt-chromium, stainless steel) per treatment, per microbe. Annotations represent the average of each bar for clarity.

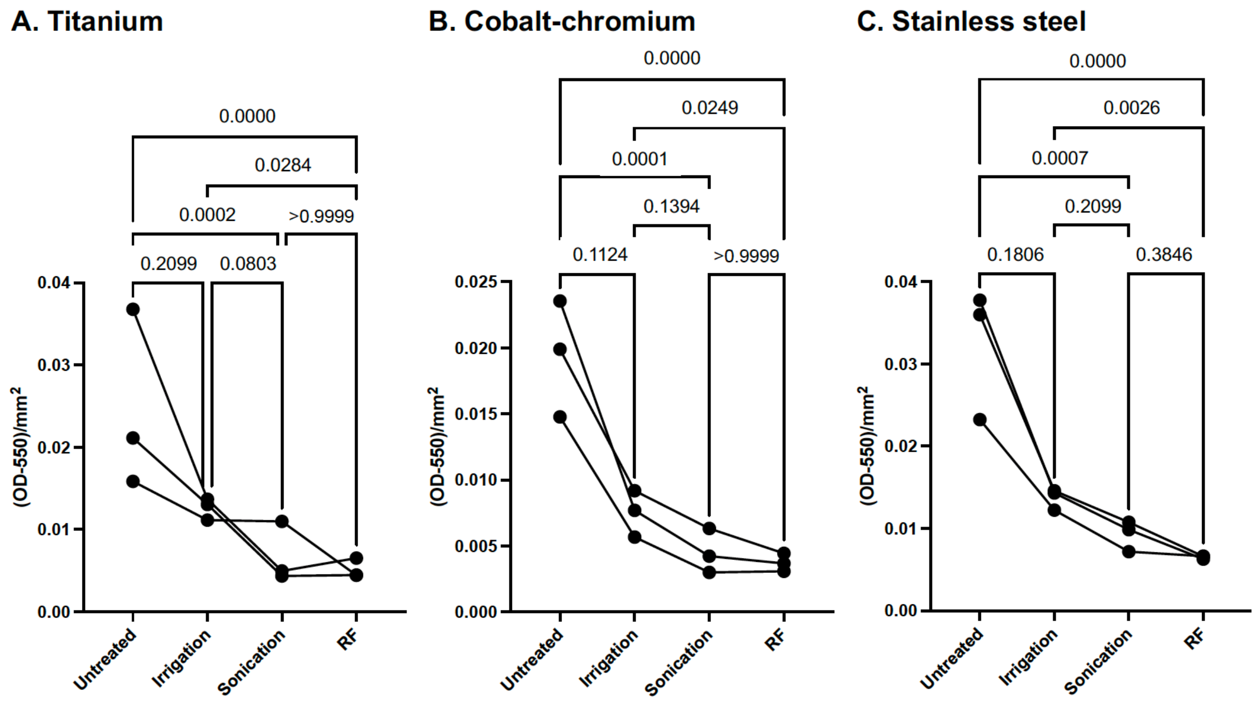

Figure S11. *Staphylococcus aureus* robust biofilms decrease following sonication and radiofrequency across all metals. *S. aureus* Newman was grown for 7 days under static conditions, with fresh medium added every 24 h, on (**A**) titanium, (**B**) cobalt-chromium and (**C**) stainless steel. Following 7-day incubation, the biomass was left untreated (Control), irrigated with PBS (Irrigation), or treated by brushing with sonication (Sonication) or brushing with sonication with radiofrequency (RF). The remaining biofilm was stained with CV, quantified and normalized to the area (mm^2) of the respective metal. Three biological replicates for each treatment group (performed in triplicate) are plotted, with lines connecting each biological replicate across all treatments. Statistical analyses of normalized biofilms were performed using Kruskal-Wallis with Dunn's multiple comparisons with a Bonferroni correction.
