# Supplementary material for: Combating orthopedic implant biofilms — SABER (Study on Agitation for Biofilm Eradication and Reduction) evaluates mechanical, sonication, and radiofrequency approaches: a preclinical in vitro study
Source: Acta Orthop. 2026 Mar 31;97:209–16. doi: 10.2340/17453674.2026.45569 (PMC13037461; doi:10.2340/17453674.2026.45569)
Supplement: Supplementary file 2 [file ActaO-97-45569-s2.pdf]

Table S1. Biofilm assay raw data and metadata.

Sample	Microbe	Metal	Treatment
Sa_titanium_unt_1a	Staphylococcus aureus	Titanium	Untreated
Sa_titanium_unt_1b	Staphylococcus aureus	Titanium	Untreated
Sa_titanium_unt_1c	Staphylococcus aureus	Titanium	Untreated
Sa_titanium_unt_2a	Staphylococcus aureus	Titanium	Untreated
Sa_titanium_unt_2b	Staphylococcus aureus	Titanium	Untreated
Sa_titanium_unt_2c	Staphylococcus aureus	Titanium	Untreated
Sa_titanium_unt_3a	Staphylococcus aureus	Titanium	Untreated
Sa_titanium_unt_3b	Staphylococcus aureus	Titanium	Untreated
Sa_titanium_unt_3c	Staphylococcus aureus	Titanium	Untreated
Sa_titanium_irr_1a	Staphylococcus aureus	Titanium	Irrigation
Sa_titanium_irr_1b	Staphylococcus aureus	Titanium	Irrigation
Sa_titanium_irr_1c	Staphylococcus aureus	Titanium	Irrigation
Sa_titanium_irr_2a	Staphylococcus aureus	Titanium	Irrigation
Sa_titanium_irr_2b	Staphylococcus aureus	Titanium	Irrigation
Sa_titanium_irr_2c	Staphylococcus aureus	Titanium	Irrigation
Sa_titanium_irr_3a	Staphylococcus aureus	Titanium	Irrigation
Sa_titanium_irr_3b	Staphylococcus aureus	Titanium	Irrigation
Sa_titanium_irr_3c	Staphylococcus aureus	Titanium	Irrigation
Sa_titanium_son_1a	Staphylococcus aureus	Titanium	Sonication
Sa_titanium_son_1b	Staphylococcus aureus	Titanium	Sonication
Sa_titanium_son_1c	Staphylococcus aureus	Titanium	Sonication
Sa_titanium_son_2a	Staphylococcus aureus	Titanium	Sonication
Sa_titanium_son_2b	Staphylococcus aureus	Titanium	Sonication
Sa_titanium_son_2c	Staphylococcus aureus	Titanium	Sonication
Sa_titanium_son_3a	Staphylococcus aureus	Titanium	Sonication
Sa_titanium_son_3b	Staphylococcus aureus	Titanium	Sonication
Sa_titanium_son_3c	Staphylococcus aureus	Titanium	Sonication
Sa_titanium_RF_1a	Staphylococcus aureus	Titanium	Radiofrequency
Sa_titanium_RF_1b	Staphylococcus aureus	Titanium	Radiofrequency
Sa_titanium_RF_1c	Staphylococcus aureus	Titanium	Radiofrequency
Sa_titanium_RF_2a	Staphylococcus aureus	Titanium	Radiofrequency
Sa_titanium_RF_2b	Staphylococcus aureus	Titanium	Radiofrequency
Sa_titanium_RF_2c	Staphylococcus aureus	Titanium	Radiofrequency
Sa_titanium_RF_3a	Staphylococcus aureus	Titanium	Radiofrequency
Sa_titanium_RF_3b	Staphylococcus aureus	Titanium	Radiofrequency
Sa_titanium_RF_3c	Staphylococcus aureus	Titanium	Radiofrequency
Sa_cc_unt_1a	Staphylococcus aureus	Cobalt-Chromium	Untreated
Sa_cc_unt_1b	Staphylococcus aureus	Cobalt-Chromium	Untreated
Sa_cc_unt_1c	Staphylococcus aureus	Cobalt-Chromium	Untreated
Sa_cc_unt_2a	Staphylococcus aureus	Cobalt-Chromium	Untreated
Sa_cc_unt_2b	Staphylococcus aureus	Cobalt-Chromium	Untreated
Sa_cc_unt_2c	Staphylococcus aureus	Cobalt-Chromium	Untreated
Sa_cc_unt_3a	Staphylococcus aureus	Cobalt-Chromium	Untreated
Sa_cc_unt_3b	Staphylococcus aureus	Cobalt-Chromium	Untreated
Sa_cc_unt_3c	Staphylococcus aureus	Cobalt-Chromium	Untreated
Sa_cc_irr_1a	Staphylococcus aureus	Cobalt-Chromium	Irrigation

Sa_cc_irr_1b	Staphylococcus aureus	Cobalt-Chromium	Irrigation
Sa_cc_irr_1c	Staphylococcus aureus	Cobalt-Chromium	Irrigation
Sa_cc_irr_2a	Staphylococcus aureus	Cobalt-Chromium	Irrigation
Sa_cc_irr_2b	Staphylococcus aureus	Cobalt-Chromium	Irrigation
Sa_cc_irr_2c	Staphylococcus aureus	Cobalt-Chromium	Irrigation
Sa_cc_irr_3a	Staphylococcus aureus	Cobalt-Chromium	Irrigation
Sa_cc_irr_3b	Staphylococcus aureus	Cobalt-Chromium	Irrigation
Sa_cc_irr_3c	Staphylococcus aureus	Cobalt-Chromium	Irrigation
Sa_cc_son_1a	Staphylococcus aureus	Cobalt-Chromium	Sonication
Sa_cc_son_1b	Staphylococcus aureus	Cobalt-Chromium	Sonication
Sa_cc_son_1c	Staphylococcus aureus	Cobalt-Chromium	Sonication
Sa_cc_son_2a	Staphylococcus aureus	Cobalt-Chromium	Sonication
Sa_cc_son_2b	Staphylococcus aureus	Cobalt-Chromium	Sonication
Sa_cc_son_2c	Staphylococcus aureus	Cobalt-Chromium	Sonication
Sa_cc_son_3a	Staphylococcus aureus	Cobalt-Chromium	Sonication
Sa_cc_son_3b	Staphylococcus aureus	Cobalt-Chromium	Sonication
Sa_cc_son_3c	Staphylococcus aureus	Cobalt-Chromium	Sonication
Sa_cc_RF_1a	Staphylococcus aureus	Cobalt-Chromium	Radiofrequency
Sa_cc_RF_1b	Staphylococcus aureus	Cobalt-Chromium	Radiofrequency
Sa_cc_RF_1c	Staphylococcus aureus	Cobalt-Chromium	Radiofrequency
Sa_cc_RF_2a	Staphylococcus aureus	Cobalt-Chromium	Radiofrequency
Sa_cc_RF_2b	Staphylococcus aureus	Cobalt-Chromium	Radiofrequency
Sa_cc_RF_2c	Staphylococcus aureus	Cobalt-Chromium	Radiofrequency
Sa_cc_RF_3a	Staphylococcus aureus	Cobalt-Chromium	Radiofrequency
Sa_cc_RF_3b	Staphylococcus aureus	Cobalt-Chromium	Radiofrequency
Sa_cc_RF_3c	Staphylococcus aureus	Cobalt-Chromium	Radiofrequency
Sa_ss_unt_1a	Staphylococcus aureus	Stainless-steel	Untreated
Sa_ss_unt_1b	Staphylococcus aureus	Stainless-steel	Untreated
Sa_ss_unt_1c	Staphylococcus aureus	Stainless-steel	Untreated
Sa_ss_unt_2a	Staphylococcus aureus	Stainless-steel	Untreated
Sa_ss_unt_2b	Staphylococcus aureus	Stainless-steel	Untreated
Sa_ss_unt_2c	Staphylococcus aureus	Stainless-steel	Untreated
Sa_ss_unt_3a	Staphylococcus aureus	Stainless-steel	Untreated
Sa_ss_unt_3b	Staphylococcus aureus	Stainless-steel	Untreated
Sa_ss_unt_3c	Staphylococcus aureus	Stainless-steel	Untreated
Sa_ss_irr_1a	Staphylococcus aureus	Stainless-steel	Irrigation
Sa_ss_irr_1b	Staphylococcus aureus	Stainless-steel	Irrigation
Sa_ss_irr_1c	Staphylococcus aureus	Stainless-steel	Irrigation
Sa_ss_irr_2a	Staphylococcus aureus	Stainless-steel	Irrigation
Sa_ss_irr_2b	Staphylococcus aureus	Stainless-steel	Irrigation
Sa_ss_irr_2c	Staphylococcus aureus	Stainless-steel	Irrigation
Sa_ss_irr_3a	Staphylococcus aureus	Stainless-steel	Irrigation
Sa_ss_irr_3b	Staphylococcus aureus	Stainless-steel	Irrigation
Sa_ss_irr_3c	Staphylococcus aureus	Stainless-steel	Irrigation
Sa_ss_son_1a	Staphylococcus aureus	Stainless-steel	Sonication
Sa_ss_son_1b	Staphylococcus aureus	Stainless-steel	Sonication
Sa_ss_son_1c	Staphylococcus aureus	Stainless-steel	Sonication
Sa_ss_son_2a	Staphylococcus aureus	Stainless-steel	Sonication

Sa_ss_son_2b	Staphylococcus aureus	Stainless-steel	Sonication
Sa_ss_son_2c	Staphylococcus aureus	Stainless-steel	Sonication
Sa_ss_son_3a	Staphylococcus aureus	Stainless-steel	Sonication
Sa_ss_son_3b	Staphylococcus aureus	Stainless-steel	Sonication
Sa_ss_son_3c	Staphylococcus aureus	Stainless-steel	Sonication
Sa_ss_RF_1a	Staphylococcus aureus	Stainless-steel	Radiofrequency
Sa_ss_RF_1b	Staphylococcus aureus	Stainless-steel	Radiofrequency
Sa_ss_RF_1c	Staphylococcus aureus	Stainless-steel	Radiofrequency
Sa_ss_RF_2a	Staphylococcus aureus	Stainless-steel	Radiofrequency
Sa_ss_RF_2b	Staphylococcus aureus	Stainless-steel	Radiofrequency
Sa_ss_RF_2c	Staphylococcus aureus	Stainless-steel	Radiofrequency
Sa_ss_RF_3a	Staphylococcus aureus	Stainless-steel	Radiofrequency
Sa_ss_RF_3b	Staphylococcus aureus	Stainless-steel	Radiofrequency
Sa_ss_RF_3c	Staphylococcus aureus	Stainless-steel	Radiofrequency
Se_titanium_unt_1a	Staphylococcus epidermidis	Titanium	Untreated
Se_titanium_unt_1b	Staphylococcus epidermidis	Titanium	Untreated
Se_titanium_unt_1c	Staphylococcus epidermidis	Titanium	Untreated
Se_titanium_unt_2a	Staphylococcus epidermidis	Titanium	Untreated
Se_titanium_unt_2b	Staphylococcus epidermidis	Titanium	Untreated
Se_titanium_unt_2c	Staphylococcus epidermidis	Titanium	Untreated
Se_titanium_unt_3a	Staphylococcus epidermidis	Titanium	Untreated
Se_titanium_unt_3b	Staphylococcus epidermidis	Titanium	Untreated
Se_titanium_unt_3c	Staphylococcus epidermidis	Titanium	Untreated
Se_titanium_irr_1a	Staphylococcus epidermidis	Titanium	Irrigation
Se_titanium_irr_1b	Staphylococcus epidermidis	Titanium	Irrigation
Se_titanium_irr_1c	Staphylococcus epidermidis	Titanium	Irrigation
Se_titanium_irr_2a	Staphylococcus epidermidis	Titanium	Irrigation
Se_titanium_irr_2b	Staphylococcus epidermidis	Titanium	Irrigation
Se_titanium_irr_2c	Staphylococcus epidermidis	Titanium	Irrigation
Se_titanium_irr_3a	Staphylococcus epidermidis	Titanium	Irrigation
Se_titanium_irr_3b	Staphylococcus epidermidis	Titanium	Irrigation
Se_titanium_irr_3c	Staphylococcus epidermidis	Titanium	Irrigation
Se_titanium_son_1a	Staphylococcus epidermidis	Titanium	Sonication
Se_titanium_son_1b	Staphylococcus epidermidis	Titanium	Sonication
Se_titanium_son_1c	Staphylococcus epidermidis	Titanium	Sonication
Se_titanium_son_2a	Staphylococcus epidermidis	Titanium	Sonication
Se_titanium_son_2b	Staphylococcus epidermidis	Titanium	Sonication
Se_titanium_son_2c	Staphylococcus epidermidis	Titanium	Sonication
Se_titanium_son_3a	Staphylococcus epidermidis	Titanium	Sonication
Se_titanium_son_3b	Staphylococcus epidermidis	Titanium	Sonication
Se_titanium_son_3c	Staphylococcus epidermidis	Titanium	Sonication
Se_titanium_RF_1a	Staphylococcus epidermidis	Titanium	Radiofrequency
Se_titanium_RF_1b	Staphylococcus epidermidis	Titanium	Radiofrequency
Se_titanium_RF_1c	Staphylococcus epidermidis	Titanium	Radiofrequency
Se_titanium_RF_2a	Staphylococcus epidermidis	Titanium	Radiofrequency
Se_titanium_RF_2b	Staphylococcus epidermidis	Titanium	Radiofrequency
Se_titanium_RF_2c	Staphylococcus epidermidis	Titanium	Radiofrequency
Se_titanium_RF_3a	Staphylococcus epidermidis	Titanium	Radiofrequency

Se_titanium_RF_3b	Staphylococcus epidermidis	Titanium	Radiofrequency
Se_titanium_RF_3c	Staphylococcus epidermidis	Titanium	Radiofrequency
Se_cc_unt_1a	Staphylococcus epidermidis	Cobalt-Chromium	Untreated
Se_cc_unt_1b	Staphylococcus epidermidis	Cobalt-Chromium	Untreated
Se_cc_unt_1c	Staphylococcus epidermidis	Cobalt-Chromium	Untreated
Se_cc_unt_2a	Staphylococcus epidermidis	Cobalt-Chromium	Untreated
Se_cc_unt_2b	Staphylococcus epidermidis	Cobalt-Chromium	Untreated
Se_cc_unt_2c	Staphylococcus epidermidis	Cobalt-Chromium	Untreated
Se_cc_unt_3a	Staphylococcus epidermidis	Cobalt-Chromium	Untreated
Se_cc_unt_3b	Staphylococcus epidermidis	Cobalt-Chromium	Untreated
Se_cc_unt_3c	Staphylococcus epidermidis	Cobalt-Chromium	Untreated
Se_cc_irr_1a	Staphylococcus epidermidis	Cobalt-Chromium	Irrigation
Se_cc_irr_1b	Staphylococcus epidermidis	Cobalt-Chromium	Irrigation
Se_cc_irr_1c	Staphylococcus epidermidis	Cobalt-Chromium	Irrigation
Se_cc_irr_2a	Staphylococcus epidermidis	Cobalt-Chromium	Irrigation
Se_cc_irr_2b	Staphylococcus epidermidis	Cobalt-Chromium	Irrigation
Se_cc_irr_2c	Staphylococcus epidermidis	Cobalt-Chromium	Irrigation
Se_cc_irr_3a	Staphylococcus epidermidis	Cobalt-Chromium	Irrigation
Se_cc_irr_3b	Staphylococcus epidermidis	Cobalt-Chromium	Irrigation
Se_cc_irr_3c	Staphylococcus epidermidis	Cobalt-Chromium	Irrigation
Se_cc_son_1a	Staphylococcus epidermidis	Cobalt-Chromium	Sonication
Se_cc_son_1b	Staphylococcus epidermidis	Cobalt-Chromium	Sonication
Se_cc_son_1c	Staphylococcus epidermidis	Cobalt-Chromium	Sonication
Se_cc_son_2a	Staphylococcus epidermidis	Cobalt-Chromium	Sonication
Se_cc_son_2b	Staphylococcus epidermidis	Cobalt-Chromium	Sonication
Se_cc_son_2c	Staphylococcus epidermidis	Cobalt-Chromium	Sonication
Se_cc_son_3a	Staphylococcus epidermidis	Cobalt-Chromium	Sonication
Se_cc_son_3b	Staphylococcus epidermidis	Cobalt-Chromium	Sonication
Se_cc_son_3c	Staphylococcus epidermidis	Cobalt-Chromium	Sonication
Se_cc_RF_1a	Staphylococcus epidermidis	Cobalt-Chromium	Radiofrequency
Se_cc_RF_1b	Staphylococcus epidermidis	Cobalt-Chromium	Radiofrequency
Se_cc_RF_1c	Staphylococcus epidermidis	Cobalt-Chromium	Radiofrequency
Se_cc_RF_2a	Staphylococcus epidermidis	Cobalt-Chromium	Radiofrequency
Se_cc_RF_2b	Staphylococcus epidermidis	Cobalt-Chromium	Radiofrequency
Se_cc_RF_2c	Staphylococcus epidermidis	Cobalt-Chromium	Radiofrequency
Se_cc_RF_3a	Staphylococcus epidermidis	Cobalt-Chromium	Radiofrequency
Se_cc_RF_3b	Staphylococcus epidermidis	Cobalt-Chromium	Radiofrequency
Se_cc_RF_3c	Staphylococcus epidermidis	Cobalt-Chromium	Radiofrequency
Se_ss_unt_1a	Staphylococcus epidermidis	Stainless-steel	Untreated
Se_ss_unt_1b	Staphylococcus epidermidis	Stainless-steel	Untreated
Se_ss_unt_1c	Staphylococcus epidermidis	Stainless-steel	Untreated
Se_ss_unt_2a	Staphylococcus epidermidis	Stainless-steel	Untreated
Se_ss_unt_2b	Staphylococcus epidermidis	Stainless-steel	Untreated
Se_ss_unt_2c	Staphylococcus epidermidis	Stainless-steel	Untreated
Se_ss_unt_3a	Staphylococcus epidermidis	Stainless-steel	Untreated
Se_ss_unt_3b	Staphylococcus epidermidis	Stainless-steel	Untreated
Se_ss_unt_3c	Staphylococcus epidermidis	Stainless-steel	Untreated
Se_ss_irr_1a	Staphylococcus epidermidis	Stainless-steel	Irrigation

Se_ss_irr_1b	Staphylococcus epidermidis	Stainless-steel	Irrigation
Se_ss_irr_1c	Staphylococcus epidermidis	Stainless-steel	Irrigation
Se_ss_irr_2a	Staphylococcus epidermidis	Stainless-steel	Irrigation
Se_ss_irr_2b	Staphylococcus epidermidis	Stainless-steel	Irrigation
Se_ss_irr_2c	Staphylococcus epidermidis	Stainless-steel	Irrigation
Se_ss_irr_3a	Staphylococcus epidermidis	Stainless-steel	Irrigation
Se_ss_irr_3b	Staphylococcus epidermidis	Stainless-steel	Irrigation
Se_ss_irr_3c	Staphylococcus epidermidis	Stainless-steel	Irrigation
Se_ss_son_1a	Staphylococcus epidermidis	Stainless-steel	Sonication
Se_ss_son_1b	Staphylococcus epidermidis	Stainless-steel	Sonication
Se_ss_son_1c	Staphylococcus epidermidis	Stainless-steel	Sonication
Se_ss_son_2a	Staphylococcus epidermidis	Stainless-steel	Sonication
Se_ss_son_2b	Staphylococcus epidermidis	Stainless-steel	Sonication
Se_ss_son_2c	Staphylococcus epidermidis	Stainless-steel	Sonication
Se_ss_son_3a	Staphylococcus epidermidis	Stainless-steel	Sonication
Se_ss_son_3b	Staphylococcus epidermidis	Stainless-steel	Sonication
Se_ss_son_3c	Staphylococcus epidermidis	Stainless-steel	Sonication
Se_ss_RF_1a	Staphylococcus epidermidis	Stainless-steel	Radiofrequency
Se_ss_RF_1b	Staphylococcus epidermidis	Stainless-steel	Radiofrequency
Se_ss_RF_1c	Staphylococcus epidermidis	Stainless-steel	Radiofrequency
Se_ss_RF_2a	Staphylococcus epidermidis	Stainless-steel	Radiofrequency
Se_ss_RF_2b	Staphylococcus epidermidis	Stainless-steel	Radiofrequency
Se_ss_RF_2c	Staphylococcus epidermidis	Stainless-steel	Radiofrequency
Se_ss_RF_3a	Staphylococcus epidermidis	Stainless-steel	Radiofrequency
Se_ss_RF_3b	Staphylococcus epidermidis	Stainless-steel	Radiofrequency
Se_ss_RF_3c	Staphylococcus epidermidis	Stainless-steel	Radiofrequency
Pa_titanium_unt_1a	Pseudomonas aeruginosa	Titanium	Untreated
Pa_titanium_unt_1b	Pseudomonas aeruginosa	Titanium	Untreated
Pa_titanium_unt_1c	Pseudomonas aeruginosa	Titanium	Untreated
Pa_titanium_unt_2a	Pseudomonas aeruginosa	Titanium	Untreated
Pa_titanium_unt_2b	Pseudomonas aeruginosa	Titanium	Untreated
Pa_titanium_unt_2c	Pseudomonas aeruginosa	Titanium	Untreated
Pa_titanium_unt_3a	Pseudomonas aeruginosa	Titanium	Untreated
Pa_titanium_unt_3b	Pseudomonas aeruginosa	Titanium	Untreated
Pa_titanium_unt_3c	Pseudomonas aeruginosa	Titanium	Untreated
Pa_titanium_irr_1a	Pseudomonas aeruginosa	Titanium	Irrigation
Pa_titanium_irr_1b	Pseudomonas aeruginosa	Titanium	Irrigation
Pa_titanium_irr_1c	Pseudomonas aeruginosa	Titanium	Irrigation
Pa_titanium_irr_2a	Pseudomonas aeruginosa	Titanium	Irrigation
Pa_titanium_irr_2b	Pseudomonas aeruginosa	Titanium	Irrigation
Pa_titanium_irr_2c	Pseudomonas aeruginosa	Titanium	Irrigation
Pa_titanium_irr_3a	Pseudomonas aeruginosa	Titanium	Irrigation
Pa_titanium_irr_3b	Pseudomonas aeruginosa	Titanium	Irrigation
Pa_titanium_irr_3c	Pseudomonas aeruginosa	Titanium	Irrigation
Pa_titanium_son_1a	Pseudomonas aeruginosa	Titanium	Sonication
Pa_titanium_son_1b	Pseudomonas aeruginosa	Titanium	Sonication
Pa_titanium_son_1c	Pseudomonas aeruginosa	Titanium	Sonication
Pa_titanium_son_2a	Pseudomonas aeruginosa	Titanium	Sonication

Pa_titanium_son_2b	Pseudomonas aeruginosa	Titanium	Sonication
Pa_titanium_son_2c	Pseudomonas aeruginosa	Titanium	Sonication
Pa_titanium_son_3a	Pseudomonas aeruginosa	Titanium	Sonication
Pa_titanium_son_3b	Pseudomonas aeruginosa	Titanium	Sonication
Pa_titanium_son_3c	Pseudomonas aeruginosa	Titanium	Sonication
Pa_titanium_RF_1a	Pseudomonas aeruginosa	Titanium	Radiofrequency
Pa_titanium_RF_1b	Pseudomonas aeruginosa	Titanium	Radiofrequency
Pa_titanium_RF_1c	Pseudomonas aeruginosa	Titanium	Radiofrequency
Pa_titanium_RF_2a	Pseudomonas aeruginosa	Titanium	Radiofrequency
Pa_titanium_RF_2b	Pseudomonas aeruginosa	Titanium	Radiofrequency
Pa_titanium_RF_2c	Pseudomonas aeruginosa	Titanium	Radiofrequency
Pa_titanium_RF_3a	Pseudomonas aeruginosa	Titanium	Radiofrequency
Pa_titanium_RF_3b	Pseudomonas aeruginosa	Titanium	Radiofrequency
Pa_titanium_RF_3c	Pseudomonas aeruginosa	Titanium	Radiofrequency
Pa_cc_unt_1a	Pseudomonas aeruginosa	Cobalt-Chromium	Untreated
Pa_cc_unt_1b	Pseudomonas aeruginosa	Cobalt-Chromium	Untreated
Pa_cc_unt_1c	Pseudomonas aeruginosa	Cobalt-Chromium	Untreated
Pa_cc_unt_2a	Pseudomonas aeruginosa	Cobalt-Chromium	Untreated
Pa_cc_unt_2b	Pseudomonas aeruginosa	Cobalt-Chromium	Untreated
Pa_cc_unt_2c	Pseudomonas aeruginosa	Cobalt-Chromium	Untreated
Pa_cc_unt_3a	Pseudomonas aeruginosa	Cobalt-Chromium	Untreated
Pa_cc_unt_3b	Pseudomonas aeruginosa	Cobalt-Chromium	Untreated
Pa_cc_unt_3c	Pseudomonas aeruginosa	Cobalt-Chromium	Untreated
Pa_cc_irr_1a	Pseudomonas aeruginosa	Cobalt-Chromium	Irrigation
Pa_cc_irr_1b	Pseudomonas aeruginosa	Cobalt-Chromium	Irrigation
Pa_cc_irr_1c	Pseudomonas aeruginosa	Cobalt-Chromium	Irrigation
Pa_cc_irr_2a	Pseudomonas aeruginosa	Cobalt-Chromium	Irrigation
Pa_cc_irr_2b	Pseudomonas aeruginosa	Cobalt-Chromium	Irrigation
Pa_cc_irr_2c	Pseudomonas aeruginosa	Cobalt-Chromium	Irrigation
Pa_cc_irr_3a	Pseudomonas aeruginosa	Cobalt-Chromium	Irrigation
Pa_cc_irr_3b	Pseudomonas aeruginosa	Cobalt-Chromium	Irrigation
Pa_cc_irr_3c	Pseudomonas aeruginosa	Cobalt-Chromium	Irrigation
Pa_cc_son_1a	Pseudomonas aeruginosa	Cobalt-Chromium	Sonication
Pa_cc_son_1b	Pseudomonas aeruginosa	Cobalt-Chromium	Sonication
Pa_cc_son_1c	Pseudomonas aeruginosa	Cobalt-Chromium	Sonication
Pa_cc_son_2a	Pseudomonas aeruginosa	Cobalt-Chromium	Sonication
Pa_cc_son_2b	Pseudomonas aeruginosa	Cobalt-Chromium	Sonication
Pa_cc_son_2c	Pseudomonas aeruginosa	Cobalt-Chromium	Sonication
Pa_cc_son_3a	Pseudomonas aeruginosa	Cobalt-Chromium	Sonication
Pa_cc_son_3b	Pseudomonas aeruginosa	Cobalt-Chromium	Sonication
Pa_cc_son_3c	Pseudomonas aeruginosa	Cobalt-Chromium	Sonication
Pa_cc_RF_1a	Pseudomonas aeruginosa	Cobalt-Chromium	Radiofrequency
Pa_cc_RF_1b	Pseudomonas aeruginosa	Cobalt-Chromium	Radiofrequency
Pa_cc_RF_1c	Pseudomonas aeruginosa	Cobalt-Chromium	Radiofrequency
Pa_cc_RF_2a	Pseudomonas aeruginosa	Cobalt-Chromium	Radiofrequency
Pa_cc_RF_2b	Pseudomonas aeruginosa	Cobalt-Chromium	Radiofrequency
Pa_cc_RF_2c	Pseudomonas aeruginosa	Cobalt-Chromium	Radiofrequency
Pa_cc_RF_3a	Pseudomonas aeruginosa	Cobalt-Chromium	Radiofrequency

Pa_cc_RF_3b	Pseudomonas aeruginosa	Cobalt-Chromium	Radiofrequency
Pa_cc_RF_3c	Pseudomonas aeruginosa	Cobalt-Chromium	Radiofrequency
Pa_ss_unt_1a	Pseudomonas aeruginosa	Stainless-steel	Untreated
Pa_ss_unt_1b	Pseudomonas aeruginosa	Stainless-steel	Untreated
Pa_ss_unt_1c	Pseudomonas aeruginosa	Stainless-steel	Untreated
Pa_ss_unt_2a	Pseudomonas aeruginosa	Stainless-steel	Untreated
Pa_ss_unt_2b	Pseudomonas aeruginosa	Stainless-steel	Untreated
Pa_ss_unt_2c	Pseudomonas aeruginosa	Stainless-steel	Untreated
Pa_ss_unt_3a	Pseudomonas aeruginosa	Stainless-steel	Untreated
Pa_ss_unt_3b	Pseudomonas aeruginosa	Stainless-steel	Untreated
Pa_ss_unt_3c	Pseudomonas aeruginosa	Stainless-steel	Untreated
Pa_ss_irr_1a	Pseudomonas aeruginosa	Stainless-steel	Irrigation
Pa_ss_irr_1b	Pseudomonas aeruginosa	Stainless-steel	Irrigation
Pa_ss_irr_1c	Pseudomonas aeruginosa	Stainless-steel	Irrigation
Pa_ss_irr_2a	Pseudomonas aeruginosa	Stainless-steel	Irrigation
Pa_ss_irr_2b	Pseudomonas aeruginosa	Stainless-steel	Irrigation
Pa_ss_irr_2c	Pseudomonas aeruginosa	Stainless-steel	Irrigation
Pa_ss_irr_3a	Pseudomonas aeruginosa	Stainless-steel	Irrigation
Pa_ss_irr_3b	Pseudomonas aeruginosa	Stainless-steel	Irrigation
Pa_ss_irr_3c	Pseudomonas aeruginosa	Stainless-steel	Irrigation
Pa_ss_son_1a	Pseudomonas aeruginosa	Stainless-steel	Sonication
Pa_ss_son_1b	Pseudomonas aeruginosa	Stainless-steel	Sonication
Pa_ss_son_1c	Pseudomonas aeruginosa	Stainless-steel	Sonication
Pa_ss_son_2a	Pseudomonas aeruginosa	Stainless-steel	Sonication
Pa_ss_son_2b	Pseudomonas aeruginosa	Stainless-steel	Sonication
Pa_ss_son_2c	Pseudomonas aeruginosa	Stainless-steel	Sonication
Pa_ss_son_3a	Pseudomonas aeruginosa	Stainless-steel	Sonication
Pa_ss_son_3b	Pseudomonas aeruginosa	Stainless-steel	Sonication
Pa_ss_son_3c	Pseudomonas aeruginosa	Stainless-steel	Sonication
Pa_ss_RF_1a	Pseudomonas aeruginosa	Stainless-steel	Radiofrequency
Pa_ss_RF_1b	Pseudomonas aeruginosa	Stainless-steel	Radiofrequency
Pa_ss_RF_1c	Pseudomonas aeruginosa	Stainless-steel	Radiofrequency
Pa_ss_RF_2a	Pseudomonas aeruginosa	Stainless-steel	Radiofrequency
Pa_ss_RF_2b	Pseudomonas aeruginosa	Stainless-steel	Radiofrequency
Pa_ss_RF_2c	Pseudomonas aeruginosa	Stainless-steel	Radiofrequency
Pa_ss_RF_3a	Pseudomonas aeruginosa	Stainless-steel	Radiofrequency
Pa_ss_RF_3b	Pseudomonas aeruginosa	Stainless-steel	Radiofrequency
Pa_ss_RF_3c	Pseudomonas aeruginosa	Stainless-steel	Radiofrequency
Ec_titanium_unt_1a	Escherichia coli	Titanium	Untreated
Ec_titanium_unt_1b	Escherichia coli	Titanium	Untreated
Ec_titanium_unt_1c	Escherichia coli	Titanium	Untreated
Ec_titanium_unt_2a	Escherichia coli	Titanium	Untreated
Ec_titanium_unt_2b	Escherichia coli	Titanium	Untreated
Ec_titanium_unt_2c	Escherichia coli	Titanium	Untreated
Ec_titanium_unt_3a	Escherichia coli	Titanium	Untreated
Ec_titanium_unt_3b	Escherichia coli	Titanium	Untreated
Ec_titanium_unt_3c	Escherichia coli	Titanium	Untreated
Ec_titanium_irr_1a	Escherichia coli	Titanium	Irrigation

Ec_titanium_irr_1b	Escherichia coli	Titanium	Irrigation
Ec_titanium_irr_1c	Escherichia coli	Titanium	Irrigation
Ec_titanium_irr_2a	Escherichia coli	Titanium	Irrigation
Ec_titanium_irr_2b	Escherichia coli	Titanium	Irrigation
Ec_titanium_irr_2c	Escherichia coli	Titanium	Irrigation
Ec_titanium_irr_3a	Escherichia coli	Titanium	Irrigation
Ec_titanium_irr_3b	Escherichia coli	Titanium	Irrigation
Ec_titanium_irr_3c	Escherichia coli	Titanium	Irrigation
Ec_titanium_son_1a	Escherichia coli	Titanium	Sonication
Ec_titanium_son_1b	Escherichia coli	Titanium	Sonication
Ec_titanium_son_1c	Escherichia coli	Titanium	Sonication
Ec_titanium_son_2a	Escherichia coli	Titanium	Sonication
Ec_titanium_son_2b	Escherichia coli	Titanium	Sonication
Ec_titanium_son_2c	Escherichia coli	Titanium	Sonication
Ec_titanium_son_3a	Escherichia coli	Titanium	Sonication
Ec_titanium_son_3b	Escherichia coli	Titanium	Sonication
Ec_titanium_son_3c	Escherichia coli	Titanium	Sonication
Ec_titanium_RF_1a	Escherichia coli	Titanium	Radiofrequency
Ec_titanium_RF_1b	Escherichia coli	Titanium	Radiofrequency
Ec_titanium_RF_1c	Escherichia coli	Titanium	Radiofrequency
Ec_titanium_RF_2a	Escherichia coli	Titanium	Radiofrequency
Ec_titanium_RF_2b	Escherichia coli	Titanium	Radiofrequency
Ec_titanium_RF_2c	Escherichia coli	Titanium	Radiofrequency
Ec_titanium_RF_3a	Escherichia coli	Titanium	Radiofrequency
Ec_titanium_RF_3b	Escherichia coli	Titanium	Radiofrequency
Ec_titanium_RF_3c	Escherichia coli	Titanium	Radiofrequency
Ec_cc_unt_1a	Escherichia coli	Cobalt-Chromium	Untreated
Ec_cc_unt_1b	Escherichia coli	Cobalt-Chromium	Untreated
Ec_cc_unt_1c	Escherichia coli	Cobalt-Chromium	Untreated
Ec_cc_unt_2a	Escherichia coli	Cobalt-Chromium	Untreated
Ec_cc_unt_2b	Escherichia coli	Cobalt-Chromium	Untreated
Ec_cc_unt_2c	Escherichia coli	Cobalt-Chromium	Untreated
Ec_cc_unt_3a	Escherichia coli	Cobalt-Chromium	Untreated
Ec_cc_unt_3b	Escherichia coli	Cobalt-Chromium	Untreated
Ec_cc_unt_3c	Escherichia coli	Cobalt-Chromium	Untreated
Ec_cc_irr_1a	Escherichia coli	Cobalt-Chromium	Irrigation
Ec_cc_irr_1b	Escherichia coli	Cobalt-Chromium	Irrigation
Ec_cc_irr_1c	Escherichia coli	Cobalt-Chromium	Irrigation
Ec_cc_irr_2a	Escherichia coli	Cobalt-Chromium	Irrigation
Ec_cc_irr_2b	Escherichia coli	Cobalt-Chromium	Irrigation
Ec_cc_irr_2c	Escherichia coli	Cobalt-Chromium	Irrigation
Ec_cc_irr_3a	Escherichia coli	Cobalt-Chromium	Irrigation
Ec_cc_irr_3b	Escherichia coli	Cobalt-Chromium	Irrigation
Ec_cc_irr_3c	Escherichia coli	Cobalt-Chromium	Irrigation
Ec_cc_son_1a	Escherichia coli	Cobalt-Chromium	Sonication
Ec_cc_son_1b	Escherichia coli	Cobalt-Chromium	Sonication
Ec_cc_son_1c	Escherichia coli	Cobalt-Chromium	Sonication
Ec_cc_son_2a	Escherichia coli	Cobalt-Chromium	Sonication

Ec_cc_son_2b	Escherichia coli	Cobalt-Chromium	Sonication
Ec_cc_son_2c	Escherichia coli	Cobalt-Chromium	Sonication
Ec_cc_son_3a	Escherichia coli	Cobalt-Chromium	Sonication
Ec_cc_son_3b	Escherichia coli	Cobalt-Chromium	Sonication
Ec_cc_son_3c	Escherichia coli	Cobalt-Chromium	Sonication
Ec_cc_RF_1a	Escherichia coli	Cobalt-Chromium	Radiofrequency
Ec_cc_RF_1b	Escherichia coli	Cobalt-Chromium	Radiofrequency
Ec_cc_RF_1c	Escherichia coli	Cobalt-Chromium	Radiofrequency
Ec_cc_RF_2a	Escherichia coli	Cobalt-Chromium	Radiofrequency
Ec_cc_RF_2b	Escherichia coli	Cobalt-Chromium	Radiofrequency
Ec_cc_RF_2c	Escherichia coli	Cobalt-Chromium	Radiofrequency
Ec_cc_RF_3a	Escherichia coli	Cobalt-Chromium	Radiofrequency
Ec_cc_RF_3b	Escherichia coli	Cobalt-Chromium	Radiofrequency
Ec_cc_RF_3c	Escherichia coli	Cobalt-Chromium	Radiofrequency
Ec_ss_unt_1a	Escherichia coli	Stainless-steel	Untreated
Ec_ss_unt_1b	Escherichia coli	Stainless-steel	Untreated
Ec_ss_unt_1c	Escherichia coli	Stainless-steel	Untreated
Ec_ss_unt_2a	Escherichia coli	Stainless-steel	Untreated
Ec_ss_unt_2b	Escherichia coli	Stainless-steel	Untreated
Ec_ss_unt_2c	Escherichia coli	Stainless-steel	Untreated
Ec_ss_unt_3a	Escherichia coli	Stainless-steel	Untreated
Ec_ss_unt_3b	Escherichia coli	Stainless-steel	Untreated
Ec_ss_unt_3c	Escherichia coli	Stainless-steel	Untreated
Ec_ss_irr_1a	Escherichia coli	Stainless-steel	Irrigation
Ec_ss_irr_1b	Escherichia coli	Stainless-steel	Irrigation
Ec_ss_irr_1c	Escherichia coli	Stainless-steel	Irrigation
Ec_ss_irr_2a	Escherichia coli	Stainless-steel	Irrigation
Ec_ss_irr_2b	Escherichia coli	Stainless-steel	Irrigation
Ec_ss_irr_2c	Escherichia coli	Stainless-steel	Irrigation
Ec_ss_irr_3a	Escherichia coli	Stainless-steel	Irrigation
Ec_ss_irr_3b	Escherichia coli	Stainless-steel	Irrigation
Ec_ss_irr_3c	Escherichia coli	Stainless-steel	Irrigation
Ec_ss_son_1a	Escherichia coli	Stainless-steel	Sonication
Ec_ss_son_1b	Escherichia coli	Stainless-steel	Sonication
Ec_ss_son_1c	Escherichia coli	Stainless-steel	Sonication
Ec_ss_son_2a	Escherichia coli	Stainless-steel	Sonication
Ec_ss_son_2b	Escherichia coli	Stainless-steel	Sonication
Ec_ss_son_2c	Escherichia coli	Stainless-steel	Sonication
Ec_ss_son_3a	Escherichia coli	Stainless-steel	Sonication
Ec_ss_son_3b	Escherichia coli	Stainless-steel	Sonication
Ec_ss_son_3c	Escherichia coli	Stainless-steel	Sonication
Ec_ss_RF_1a	Escherichia coli	Stainless-steel	Radiofrequency
Ec_ss_RF_1b	Escherichia coli	Stainless-steel	Radiofrequency
Ec_ss_RF_1c	Escherichia coli	Stainless-steel	Radiofrequency
Ec_ss_RF_2a	Escherichia coli	Stainless-steel	Radiofrequency
Ec_ss_RF_2b	Escherichia coli	Stainless-steel	Radiofrequency
Ec_ss_RF_2c	Escherichia coli	Stainless-steel	Radiofrequency
Ec_ss_RF_3a	Escherichia coli	Stainless-steel	Radiofrequency

Ec_ss_RF_3b	Escherichia coli	Stainless-steel	Radiofrequency
Ec_ss_RF_3c	Escherichia coli	Stainless-steel	Radiofrequency

Replicate	OD550	Metal_Size (mm2)	OD_norm
Sa_titanium_unt_1	0,5405	64	0,00844531
Sa_titanium_unt_1	0,7292	64	0,01139375
Sa_titanium_unt_1	0,7624	64	0,0119125
Sa_titanium_unt_2	2,234	64	0,03490625
Sa_titanium_unt_2	1,8771	64	0,02932969
Sa_titanium_unt_2	2,0919	64	0,03268594
Sa_titanium_unt_3	2,2857	64	0,03571406
Sa_titanium_unt_3	1,9325	64	0,03019531
Sa_titanium_unt_3	1,9526	64	0,03050938
Sa_titanium_irr_1	0,1642	64	0,00256563
Sa_titanium_irr_1	0,4916	64	0,00768125
Sa_titanium_irr_1	1,2796	64	0,01999375
Sa_titanium_irr_2	0,1657	64	0,00258906
Sa_titanium_irr_2	0,1555	64	0,00242969
Sa_titanium_irr_2	0,2397	64	0,00374531
Sa_titanium_irr_3	0,1175	64	0,00183594
Sa_titanium_irr_3	0,2607	64	0,00407344
Sa_titanium_irr_3	0,1183	64	0,00184844
Sa_titanium_son_1	0,2189	64	0,00342031
Sa_titanium_son_1	0,172	64	0,0026875
Sa_titanium_son_1	0,6583	64	0,01028594
Sa_titanium_son_2	0,1728	64	0,0027
Sa_titanium_son_2	0,188	64	0,0029375
Sa_titanium_son_2	0,1282	64	0,00200313
Sa_titanium_son_3	0,1615	64	0,00252344
Sa_titanium_son_3	0,1043	64	0,00162969
Sa_titanium_son_3	0,0938	64	0,00146563
Sa_titanium_RF_1	0,0994	64	0,00155313
Sa_titanium_RF_1	0,2406	64	0,00375938
Sa_titanium_RF_1	0,1406	64	0,00219688
Sa_titanium_RF_2	0,1101	64	0,00172031
Sa_titanium_RF_2	0,122	64	0,00190625
Sa_titanium_RF_2	0,1525	64	0,00238281
Sa_titanium_RF_3	0,0953	64	0,00148906
Sa_titanium_RF_3	0,1086	64	0,00169688
Sa_titanium_RF_3	0,0856	64	0,0013375
Sa_cc_unt_1	2,5626	108,8	0,02355331
Sa_cc_unt_1	2,3431	108,8	0,02153585
Sa_cc_unt_1	2,053	108,8	0,01886949
Sa_cc_unt_2	2,3028	108,8	0,02116544
Sa_cc_unt_2	1,2223	108,8	0,01123438
Sa_cc_unt_2	1,4429	108,8	0,01326195
Sa_cc_unt_3	2,4536	108,8	0,02255147
Sa_cc_unt_3	2,4876	108,8	0,02286397
Sa_cc_unt_3	2,5868	108,8	0,02377574
Sa_cc_irr_1	0,2229	108,8	0,00204871

Sa_cc_irr_1	0,1845	108,8	0,00169577
Sa_cc_irr_1	0,1385	108,8	0,00127298
Sa_cc_irr_2	0,0675	108,8	0,0006204
Sa_cc_irr_2	0,0822	108,8	0,00075551
Sa_cc_irr_2	0,1136	108,8	0,00104412
Sa_cc_irr_3	0,0796	108,8	0,00073162
Sa_cc_irr_3	0,0766	108,8	0,00070404
Sa_cc_irr_3	0,1042	108,8	0,00095772
Sa_cc_son_1	0,0988	108,8	0,00090809
Sa_cc_son_1	0,1051	108,8	0,00096599
Sa_cc_son_1	0,0682	108,8	0,00062684
Sa_cc_son_2	0,0382	108,8	0,0003511
Sa_cc_son_2	0,0419	108,8	0,00038511
Sa_cc_son_2	0,0844	108,8	0,00077574
Sa_cc_son_3	0,0542	108,8	0,00049816
Sa_cc_son_3	0,0732	108,8	0,00067279
Sa_cc_son_3	0,0809	108,8	0,00074357
Sa_cc_RF_1	0,0575	108,8	0,00052849
Sa_cc_RF_1	0,0631	108,8	0,00057996
Sa_cc_RF_1	0,0606	108,8	0,00055699
Sa_cc_RF_2	0,0409	108,8	0,00037592
Sa_cc_RF_2	0,0424	108,8	0,00038971
Sa_cc_RF_2	0,0623	108,8	0,00057261
Sa_cc_RF_3	0,0573	108,8	0,00052665
Sa_cc_RF_3	0,0656	108,8	0,00060294
Sa_cc_RF_3	0,0522	108,8	0,00047978
Sa_ss_unt_1	1,4305	66	0,02167424
Sa_ss_unt_1	0,696	66	0,01054545
Sa_ss_unt_1	1,4015	66	0,02123485
Sa_ss_unt_2	1,511	66	0,02289394
Sa_ss_unt_2	1,3088	66	0,0198303
Sa_ss_unt_2	1,4407	66	0,02182879
Sa_ss_unt_3	1,348	66	0,02042424
Sa_ss_unt_3	1,2353	66	0,01871667
Sa_ss_unt_3	1,8521	66	0,02806212
Sa_ss_irr_1	0,0895	66	0,00135606
Sa_ss_irr_1	0,0988	66	0,00149697
Sa_ss_irr_1	0,1298	66	0,00196667
Sa_ss_irr_2	0,2394	66	0,00362727
Sa_ss_irr_2	0,1925	66	0,00291667
Sa_ss_irr_2	0,0552	66	0,00083636
Sa_ss_irr_3	0,0453	66	0,00068636
Sa_ss_irr_3	0,1001	66	0,00151667
Sa_ss_irr_3	0,1002	66	0,00151818
Sa_ss_son_1	0,0808	66	0,00122424
Sa_ss_son_1	0,0617	66	0,00093485
Sa_ss_son_1	0,0438	66	0,00066364
Sa_ss_son_2	0,0586	66	0,00088788

Sa_ss_son_2	0,0482	66	0,0007303
Sa_ss_son_2	0,0344	66	0,00052121
Sa_ss_son_3	0,0673	66	0,0010197
Sa_ss_son_3	0,0471	66	0,00071364
Sa_ss_son_3	0,0712	66	0,00107879
Sa_ss_RF_1	0,0269	66	0,00040758
Sa_ss_RF_1	0,0378	66	0,00057273
Sa_ss_RF_1	0,0363	66	0,00055
Sa_ss_RF_2	0,0465	66	0,00070455
Sa_ss_RF_2	0,0413	66	0,00062576
Sa_ss_RF_2	0,0355	66	0,00053788
Sa_ss_RF_3	0,0368	66	0,00055758
Sa_ss_RF_3	0,0531	66	0,00080455
Sa_ss_RF_3	0,0641	66	0,00097121
Se_titanium_unt_1	2,0456	64	0,0319625
Se_titanium_unt_1	2,3818	64	0,03721563
Se_titanium_unt_1	2,2243	64	0,03475469
Se_titanium_unt_2	2,5716	64	0,04018125
Se_titanium_unt_2	2,4616	64	0,0384625
Se_titanium_unt_2	2,5668	64	0,04010625
Se_titanium_unt_3	2,3967	64	0,03744844
Se_titanium_unt_3	2,468	64	0,0385625
Se_titanium_unt_3	2,4763	64	0,03869219
Se_titanium_irr_1	2,1407	64	0,03344844
Se_titanium_irr_1	2,1435	64	0,03349219
Se_titanium_irr_1	2,1192	64	0,0331125
Se_titanium_irr_2	2,0065	64	0,03135156
Se_titanium_irr_2	1,8227	64	0,02847969
Se_titanium_irr_2	2,0492	64	0,03201875
Se_titanium_irr_3	1,9526	64	0,03050938
Se_titanium_irr_3	2,2415	64	0,03502344
Se_titanium_irr_3	2,2011	64	0,03439219
Se_titanium_son_1	0,4013	64	0,00627031
Se_titanium_son_1	0,2236	64	0,00349375
Se_titanium_son_1	0,246	64	0,00384375
Se_titanium_son_2	0,7349	64	0,01148281
Se_titanium_son_2	1,0652	64	0,01664375
Se_titanium_son_2	1,6261	64	0,02540781
Se_titanium_son_3	1,1715	64	0,01830469
Se_titanium_son_3	0,5079	64	0,00793594
Se_titanium_son_3	0,9166	64	0,01432188
Se_titanium_RF_1	0,1732	64	0,00270625
Se_titanium_RF_1	0,1866	64	0,00291563
Se_titanium_RF_1	0,1626	64	0,00254063
Se_titanium_RF_2	0,5255	64	0,00821094
Se_titanium_RF_2	0,6667	64	0,01041719
Se_titanium_RF_2	0,5977	64	0,00933906
Se_titanium_RF_3	1,8204	64	0,02844375

Se_titanium_RF_3	0,3366	64	0,00525938
Se_titanium_RF_3	0,388	64	0,0060625
Se_cc_unt_1	2,5401	108,8	0,02334651
Se_cc_unt_1	2,5471	108,8	0,02341085
Se_cc_unt_1	2,5611	108,8	0,02353952
Se_cc_unt_2	2,6547	108,8	0,02439982
Se_cc_unt_2	2,3632	108,8	0,02172059
Se_cc_unt_2	2,698	108,8	0,02479779
Se_cc_unt_3	2,6491	108,8	0,02434835
Se_cc_unt_3	2,5454	108,8	0,02339522
Se_cc_unt_3	2,5654	108,8	0,02357904
Se_cc_irr_1	2,5352	108,8	0,02330147
Se_cc_irr_1	2,5028	108,8	0,02300368
Se_cc_irr_1	2,5554	108,8	0,02348713
Se_cc_irr_2	2,4393	108,8	0,02242004
Se_cc_irr_2	2,0661	108,8	0,01898989
Se_cc_irr_2	2,0147	108,8	0,01851746
Se_cc_irr_3	2,4283	108,8	0,02231893
Se_cc_irr_3	2,4904	108,8	0,02288971
Se_cc_irr_3	2,4291	108,8	0,02232629
Se_cc_son_1	0,4517	108,8	0,00415165
Se_cc_son_1	0,8418	108,8	0,00773713
Se_cc_son_1	0,8478	108,8	0,00779228
Se_cc_son_2	1,092	108,8	0,01003676
Se_cc_son_2	1,3971	108,8	0,01284099
Se_cc_son_2	1,3996	108,8	0,01286397
Se_cc_son_3	1,0878	108,8	0,00999816
Se_cc_son_3	0,7666	108,8	0,00704596
Se_cc_son_3	0,9768	108,8	0,00897794
Se_cc_RF_1	0,4959	108,8	0,0045579
Se_cc_RF_1	0,4736	108,8	0,00435294
Se_cc_RF_1	0,3105	108,8	0,00285386
Se_cc_RF_2	1,5891	108,8	0,0146057
Se_cc_RF_2	1,7225	108,8	0,0158318
Se_cc_RF_2	1,678	108,8	0,01542279
Se_cc_RF_3	1,0646	108,8	0,00978493
Se_cc_RF_3	0,9808	108,8	0,00901471
Se_cc_RF_3	0,5438	108,8	0,00499816
Se_ss_unt_1	1,7915	66	0,02714394
Se_ss_unt_1	1,7239	66	0,0261197
Se_ss_unt_1	2,3219	66	0,0351803
Se_ss_unt_2	2,2629	66	0,03428636
Se_ss_unt_2	2,4343	66	0,03688333
Se_ss_unt_2	2,4127	66	0,03655606
Se_ss_unt_3	2,6254	66	0,03977879
Se_ss_unt_3	2,5061	66	0,03797121
Se_ss_unt_3	2,5888	66	0,03922424
Se_ss_irr_1	2,2325	66	0,03382576

Se_ss_irr_1	2,0917	66	0,03169242
Se_ss_irr_1	2,3573	66	0,03571667
Se_ss_irr_2	1,1442	66	0,01733636
Se_ss_irr_2	1,1539	66	0,01748333
Se_ss_irr_2	2,356	66	0,03569697
Se_ss_irr_3	2,7116	66	0,04108485
Se_ss_irr_3	2,5108	66	0,03804242
Se_ss_irr_3	2,5081	66	0,03800152
Se_ss_son_1	0,3746	66	0,00567576
Se_ss_son_1	0,2266	66	0,00343333
Se_ss_son_1	0,339	66	0,00513636
Se_ss_son_2	1,4688	66	0,02225455
Se_ss_son_2	1,3699	66	0,02075606
Se_ss_son_2	1,1491	66	0,01741061
Se_ss_son_3	1,5799	66	0,02393788
Se_ss_son_3	1,4912	66	0,02259394
Se_ss_son_3	1,9016	66	0,02881212
Se_ss_RF_1	0,521	66	0,00789394
Se_ss_RF_1	0,2305	66	0,00349242
Se_ss_RF_1	0,363	66	0,0055
Se_ss_RF_2	0,8256	66	0,01250909
Se_ss_RF_2	0,85	66	0,01287879
Se_ss_RF_2	0,5497	66	0,00832879
Se_ss_RF_3	1,513	66	0,02292424
Se_ss_RF_3	1,6979	66	0,02572576
Se_ss_RF_3	1,011	66	0,01531818
Pa_titanium_unt_1	2,4008	64	0,0375125
Pa_titanium_unt_1	2,4285	64	0,03794531
Pa_titanium_unt_1	2,22	64	0,0346875
Pa_titanium_unt_2	1,7272	64	0,0269875
Pa_titanium_unt_2	2,4286	64	0,03794688
Pa_titanium_unt_2	2,308	64	0,0360625
Pa_titanium_unt_3	1,1627	64	0,01816719
Pa_titanium_unt_3	1,2627	64	0,01972969
Pa_titanium_unt_3	1,0012	64	0,01564375
Pa_titanium_irr_1	0,4638	64	0,00724688
Pa_titanium_irr_1	0,5111	64	0,00798594
Pa_titanium_irr_1	0,2615	64	0,00408594
Pa_titanium_irr_2	0,4873	64	0,00761406
Pa_titanium_irr_2	0,5474	64	0,00855313
Pa_titanium_irr_2	0,4526	64	0,00707188
Pa_titanium_irr_3	0,4441	64	0,00693906
Pa_titanium_irr_3	0,6105	64	0,00953906
Pa_titanium_irr_3	1,07	64	0,01671875
Pa_titanium_son_1	0,5264	64	0,008225
Pa_titanium_son_1	0,4412	64	0,00689375
Pa_titanium_son_1	0,3354	64	0,00524063
Pa_titanium_son_2	0,3677	64	0,00574531

Pa_titanium_son_2	0,2167	64	0,00338594
Pa_titanium_son_2	0,2743	64	0,00428594
Pa_titanium_son_3	0,3246	64	0,00507188
Pa_titanium_son_3	0,2701	64	0,00422031
Pa_titanium_son_3	0,3324	64	0,00519375
Pa_titanium_RF_1	0,4837	64	0,00755781
Pa_titanium_RF_1	0,5375	64	0,00839844
Pa_titanium_RF_1	0,5584	64	0,008725
Pa_titanium_RF_2	0,5012	64	0,00783125
Pa_titanium_RF_2	0,222	64	0,00346875
Pa_titanium_RF_2	0,3795	64	0,00592969
Pa_titanium_RF_3	0,3253	64	0,00508281
Pa_titanium_RF_3	0,3406	64	0,00532188
Pa_titanium_RF_3	0,2331	64	0,00364219
Pa_cc_unt_1	2,6706	108,8	0,02454596
Pa_cc_unt_1	2,5417	108,8	0,02336121
Pa_cc_unt_1	2,6346	108,8	0,02421507
Pa_cc_unt_2	1,9484	108,8	0,01790809
Pa_cc_unt_2	2,4271	108,8	0,0223079
Pa_cc_unt_2	2,2182	108,8	0,02038787
Pa_cc_unt_3	1,7024	108,8	0,01564706
Pa_cc_unt_3	1,4035	108,8	0,01289982
Pa_cc_unt_3	1,1465	108,8	0,01053768
Pa_cc_irr_1	0,6709	108,8	0,00616636
Pa_cc_irr_1	0,5298	108,8	0,00486949
Pa_cc_irr_1	0,584	108,8	0,00536765
Pa_cc_irr_2	0,4073	108,8	0,00374357
Pa_cc_irr_2	0,4541	108,8	0,00417371
Pa_cc_irr_2	0,4332	108,8	0,00398162
Pa_cc_irr_3	0,5871	108,8	0,00539614
Pa_cc_irr_3	0,4757	108,8	0,00437224
Pa_cc_irr_3	0,6755	108,8	0,00620864
Pa_cc_son_1	1,4247	108,8	0,01309467
Pa_cc_son_1	0,8549	108,8	0,00785754
Pa_cc_son_1	0,672	108,8	0,00617647
Pa_cc_son_2	0,5115	108,8	0,00470129
Pa_cc_son_2	1,0263	108,8	0,0094329
Pa_cc_son_2	0,5759	108,8	0,0052932
Pa_cc_son_3	0,8233	108,8	0,0075671
Pa_cc_son_3	0,3063	108,8	0,00281526
Pa_cc_son_3	0,6699	108,8	0,00615717
Pa_cc_RF_1	0,6707	108,8	0,00616452
Pa_cc_RF_1	0,7795	108,8	0,00716452
Pa_cc_RF_1	0,4573	108,8	0,00420313
Pa_cc_RF_2	0,4979	108,8	0,00457629
Pa_cc_RF_2	0,242	108,8	0,00222426
Pa_cc_RF_2	0,1935	108,8	0,00177849
Pa_cc_RF_3	0,2893	108,8	0,00265901

Pa_cc_RF_3	0,3452	108,8	0,00317279
Pa_cc_RF_3	0,2269	108,8	0,00208548
Pa_ss_unt_1	2,6737	66	0,04051061
Pa_ss_unt_1	2,3649	66	0,03583182
Pa_ss_unt_1	2,3518	66	0,03563333
Pa_ss_unt_2	1,7279	66	0,0261803
Pa_ss_unt_2	2,059	66	0,03119697
Pa_ss_unt_2	2,3506	66	0,03561515
Pa_ss_unt_3	0,834	66	0,01263636
Pa_ss_unt_3	0,7084	66	0,01073333
Pa_ss_unt_3	0,3892	66	0,00589697
Pa_ss_irr_1	0,4304	66	0,00652121
Pa_ss_irr_1	0,4886	66	0,00740303
Pa_ss_irr_1	0,674	66	0,01021212
Pa_ss_irr_2	0,7838	66	0,01187576
Pa_ss_irr_2	0,4479	66	0,00678636
Pa_ss_irr_2	0,5039	66	0,00763485
Pa_ss_irr_3	0,3815	66	0,0057803
Pa_ss_irr_3	0,5462	66	0,00827576
Pa_ss_irr_3	0,6363	66	0,00964091
Pa_ss_son_1	0,4555	66	0,00690152
Pa_ss_son_1	0,7297	66	0,01105606
Pa_ss_son_1	0,5685	66	0,00861364
Pa_ss_son_2	0,6517	66	0,00987424
Pa_ss_son_2	0,3834	66	0,00580909
Pa_ss_son_2	0,3133	66	0,00474697
Pa_ss_son_3	0,4479	66	0,00678636
Pa_ss_son_3	0,7466	66	0,01131212
Pa_ss_son_3	0,562	66	0,00851515
Pa_ss_RF_1	0,7756	66	0,01175152
Pa_ss_RF_1	0,8209	66	0,01243788
Pa_ss_RF_1	0,4043	66	0,00612576
Pa_ss_RF_2	0,5167	66	0,00782879
Pa_ss_RF_2	0,3472	66	0,00526061
Pa_ss_RF_2	0,4892	66	0,00741212
Pa_ss_RF_3	0,1928	66	0,00292121
Pa_ss_RF_3	0,3897	66	0,00590455
Pa_ss_RF_3	0,2479	66	0,00375606
Ec_titanium_unt_1	0,6983	64	0,01091094
Ec_titanium_unt_1	0,648	64	0,010125
Ec_titanium_unt_1	0,5931	64	0,00926719
Ec_titanium_unt_2	0,4173	64	0,00652031
Ec_titanium_unt_2	0,5479	64	0,00856094
Ec_titanium_unt_2	0,5942	64	0,00928438
Ec_titanium_unt_3	0,9098	64	0,01421563
Ec_titanium_unt_3	0,4956	64	0,00774375
Ec_titanium_unt_3	0,4897	64	0,00765156
Ec_titanium_irr_1	0,4296	64	0,0067125

Ec_titanium_irr_1	0,3523	64	0,00550469
Ec_titanium_irr_1	0,4832	64	0,00755
Ec_titanium_irr_2	0,3681	64	0,00575156
Ec_titanium_irr_2	0,1469	64	0,00229531
Ec_titanium_irr_2	0,6858	64	0,01071563
Ec_titanium_irr_3	0,3836	64	0,00599375
Ec_titanium_irr_3	0,499	64	0,00779688
Ec_titanium_irr_3	0,2187	64	0,00341719
Ec_titanium_son_1	0,1432	64	0,0022375
Ec_titanium_son_1	0,1473	64	0,00230156
Ec_titanium_son_1	0,1276	64	0,00199375
Ec_titanium_son_2	0,1884	64	0,00294375
Ec_titanium_son_2	0,1468	64	0,00229375
Ec_titanium_son_2	0,1288	64	0,0020125
Ec_titanium_son_3	0,2081	64	0,00325156
Ec_titanium_son_3	0,2601	64	0,00406406
Ec_titanium_son_3	0,1823	64	0,00284844
Ec_titanium_RF_1	0,1639	64	0,00256094
Ec_titanium_RF_1	0,2284	64	0,00356875
Ec_titanium_RF_1	0,1406	64	0,00219688
Ec_titanium_RF_2	0,0991	64	0,00154844
Ec_titanium_RF_2	0,0961	64	0,00150156
Ec_titanium_RF_2	0,0905	64	0,00141406
Ec_titanium_RF_3	0,2358	64	0,00368438
Ec_titanium_RF_3	0,2628	64	0,00410625
Ec_titanium_RF_3	0,2485	64	0,00388281
Ec_cc_unt_1	0,9333	108,8	0,00857813
Ec_cc_unt_1	0,8032	108,8	0,00738235
Ec_cc_unt_1	0,7538	108,8	0,00692831
Ec_cc_unt_2	1,6449	108,8	0,01511857
Ec_cc_unt_2	1,035	108,8	0,00951287
Ec_cc_unt_2	1,1339	108,8	0,01042188
Ec_cc_unt_3	0,7994	108,8	0,00734743
Ec_cc_unt_3	1,1414	108,8	0,01049081
Ec_cc_unt_3	0,6938	108,8	0,00637684
Ec_cc_irr_1	0,1755	108,8	0,00161305
Ec_cc_irr_1	0,1986	108,8	0,00182537
Ec_cc_irr_1	0,3091	108,8	0,00284099
Ec_cc_irr_2	0,3059	108,8	0,00281158
Ec_cc_irr_2	0,3669	108,8	0,00337224
Ec_cc_irr_2	0,4576	108,8	0,00420588
Ec_cc_irr_3	0,193	108,8	0,0017739
Ec_cc_irr_3	0,1646	108,8	0,00151287
Ec_cc_irr_3	0,1762	108,8	0,00161949
Ec_cc_son_1	0,1952	108,8	0,00179412
Ec_cc_son_1	0,2673	108,8	0,0024568
Ec_cc_son_1	0,2275	108,8	0,00209099
Ec_cc_son_2	0,2537	108,8	0,0023318

Ec_cc_son_2	0,1515	108,8	0,00139246
Ec_cc_son_2	0,2319	108,8	0,00213143
Ec_cc_son_3	0,2342	108,8	0,00215257
Ec_cc_son_3	0,2456	108,8	0,00225735
Ec_cc_son_3	0,1425	108,8	0,00130974
Ec_cc_RF_1	0,1862	108,8	0,0017114
Ec_cc_RF_1	0,1363	108,8	0,00125276
Ec_cc_RF_1	0,1139	108,8	0,00104688
Ec_cc_RF_2	0,1265	108,8	0,00116268
Ec_cc_RF_2	0,1351	108,8	0,00124173
Ec_cc_RF_2	0,1725	108,8	0,00158548
Ec_cc_RF_3	0,1764	108,8	0,00162132
Ec_cc_RF_3	0,1959	108,8	0,00180055
Ec_cc_RF_3	0,1945	108,8	0,00178768
Ec_ss_unt_1	0,5866	66	0,00888788
Ec_ss_unt_1	0,7907	66	0,0119803
Ec_ss_unt_1	0,574	66	0,00869697
Ec_ss_unt_2	0,8241	66	0,01248636
Ec_ss_unt_2	1,0549	66	0,01598333
Ec_ss_unt_2	1,0806	66	0,01637273
Ec_ss_unt_3	0,693	66	0,0105
Ec_ss_unt_3	0,6381	66	0,00966818
Ec_ss_unt_3	0,6978	66	0,01057273
Ec_ss_irr_1	0,1537	66	0,00232879
Ec_ss_irr_1	0,1968	66	0,00298182
Ec_ss_irr_1	0,2096	66	0,00317576
Ec_ss_irr_2	0,3372	66	0,00510909
Ec_ss_irr_2	0,3481	66	0,00527424
Ec_ss_irr_2	1,0138	66	0,01536061
Ec_ss_irr_3	0,2354	66	0,00356667
Ec_ss_irr_3	0,2628	66	0,00398182
Ec_ss_irr_3	0,4275	66	0,00647727
Ec_ss_son_1	0,1315	66	0,00199242
Ec_ss_son_1	0,1585	66	0,00240152
Ec_ss_son_1	0,2043	66	0,00309545
Ec_ss_son_2	0,2845	66	0,00431061
Ec_ss_son_2	0,2838	66	0,0043
Ec_ss_son_2	0,2576	66	0,00390303
Ec_ss_son_3	0,214	66	0,00324242
Ec_ss_son_3	0,2669	66	0,00404394
Ec_ss_son_3	0,2421	66	0,00366818
Ec_ss_RF_1	0,1242	66	0,00188182
Ec_ss_RF_1	0,1799	66	0,00272576
Ec_ss_RF_1	0,1447	66	0,00219242
Ec_ss_RF_2	0,2571	66	0,00389545
Ec_ss_RF_2	0,1855	66	0,00281061
Ec_ss_RF_2	0,1583	66	0,00239848
Ec_ss_RF_3	0,1502	66	0,00227576

Ec_ss_RF_3	0,1454	66	0,00220303
Ec_ss_RF_3	0,1612	66	0,00244242
