# Supplementary material for: Combating orthopedic implant biofilms — SABER (Study on Agitation for Biofilm Eradication and Reduction) evaluates mechanical, sonication, and radiofrequency approaches: a preclinical in vitro study
Source: Acta Orthop. 2026 Mar 31;97:209–16. doi: 10.2340/17453674.2026.45569 (PMC13037461; doi:10.2340/17453674.2026.45569)
Supplement: Supplementary file 3 [file ActaO-97-45569-s3.pdf]

Supplemental Tables 2-35. Summary of statistical analyses.

Supplemental Table 2. One-way ANOVA with Tukey's multiple comparisons test of *S. aureus* 24h on titanium

Tukey's multiple comparisons test	Mean Diff.	95% CI of diff.	Below threshold?	Summary	Adj. P value
Untreated vs. Irrigation	0,01981	0.002376 to 0.03725	Yes	*	0,0272
Untreated vs. Sonication	0,02172	0.004278 to 0.03915	Yes	*	0,0169
Untreated vs. RF	0,02301	0.005568 to 0.04044	Yes	*	0,0124
Irrigation vs. Sonication	0,001901	-0.01554 to 0.01934	No	ns	0,9843
Irrigation vs. RF	0,003191	-0.01425 to 0.02063	No	ns	0,9335
Sonication vs. RF	0,00129	-0.01615 to 0.01873	No	ns	0,9949

Supplemental Table 3. One-way ANOVA with Tukey's multiple comparisons test of *S. aureus* 24h on cobalt-chromium

Tukey's multiple comparisons test	Mean Diff.	95% CI of diff.	Below threshold?	Summary	Adj. P value
Untreated vs. Irrigation	0,01878	0.01335 to 0.02420	Yes	****	<0.0001
Untreated vs. Sonication	0,01921	0.01378 to 0.02464	Yes	****	<0.0001
Untreated vs. RF	0,01936	0.01393 to 0.02478	Yes	****	<0.0001
Irrigation vs. Sonication	0,0004337	-0.004995 to 0.005862	No	ns	0,9937
Irrigation vs. RF	0,0005798	-0.004849 to 0.006008	No	ns	0,9852
Sonication vs. RF	0,000146	-0.005283 to 0.005575	No	ns	0,9998

Supplemental Table 4. One-way ANOVA with Tukey's multiple comparisons test of *S. aureus* 24h on stainless steel

Tukey's multiple comparisons test	Mean Diff.	95% CI of diff.	Below threshold?	Summary	Adj. P value
Untreated vs. Irrigation	0,01881	0.01552 to 0.02210	Yes	****	<0.0001
Untreated vs. Sonication	0,01972	0.01642 to 0.02301	Yes	****	<0.0001
Untreated vs. RF	0,01994	0.01665 to 0.02323	Yes	****	<0.0001
Irrigation vs. Sonication	0,0009052	-0.002386 to 0.004197	No	ns	0,8149
Irrigation vs. RF	0,001132	-0.002159 to 0.004424	No	ns	0,6986
Sonication vs. RF	0,0002269	-0.003064 to 0.003518	No	ns	0,9959

Supplemental Table 5. Kruskal-Wallis with Dunn's multiple comparisons test with a Bonferroni correction of *S. aureus* 24h on titanium

Dunn's multiple comparisons test	Dunn's compa	Significant?	Summary	Adj. P value
Untreated vs. Irrigation	-2,662252	Yes	*	0,0233
Untreated vs. Sonication	-3,266292	Yes	***	0,0033
Untreated vs. RF	-4,586233	Yes	****	0,0000
Irrigation vs. Sonication	-0,60404	No	ns	>0.9999

Irrigation vs. RF	-1,92398	No	ns	0,1631
Sonication vs. RF	-1,31994	No	ns	0,5606

Supplemental Table 6. Kruskal-Wallis with Dunn's multiple comparisons test with a Bonferroni correction of *S. aureus* 24h on cobalt-chro

Dunn's multiple comparisons test	Dunn's compa	Significant?	Summary	Adj. P value
Untreated vs. Irrigation	-2,214814	No	ns	0,0803
Untreated vs. Sonication	-3,736102	Yes	****	0,0006
Untreated vs. RF	-4,921811	Yes	****	0,0000
Irrigation vs. Sonication	-1,521287	No	ns	0,3846
Irrigation vs. RF	-2,706996	Yes	*	0,0204
Sonication vs. RF	-1,185709	No	ns	0,7072

Supplemental Table 7. Kruskal-Wallis with Dunn's multiple comparisons test with a Bonferroni correction of *S. aureus* 24h on stainless st

Dunn's multiple comparisons test	Dunn's compa	Significant?	Summary	Adj. P value
Untreated vs. Irrigation	-2,170071	No	ns	0,09
Untreated vs. Sonication	-3,736102	Yes	****	0,0006
Untreated vs. RF	-4,966554	Yes	****	0,0000
Irrigation vs. Sonication	-1,56603	No	ns	0,352
Irrigation vs. RF	-2,796483	Yes	*	0,0155
Sonication vs. RF	-1,230452	No	ns	0,6556

Supplemental Table 8. One-way ANOVA with Tukey's multiple comparisons test of *S. epidermidis* 24h on titanium

Tukey's multiple comparisons test	Mean Diff.	95% CI of diff.	Below threshold?	Summary	Adj. P value
Untreated vs. Irrigation	0,005062	-0.006877 to 0.01700	No	ns	0,5559
Untreated vs. Sonication	0,02552	0.01358 to 0.03746	Yes	***	0,0006
Untreated vs. RF	0,02905	0.01712 to 0.04099	Yes	***	0,0002
Irrigation vs. Sonication	0,02046	0.008519 to 0.03240	Yes	**	0,0026
Irrigation vs. RF	0,02399	0.01205 to 0.03593	Yes	***	0,0009
Sonication vs. RF	0,003534	-0.008405 to 0.01547	No	ns	0,7811

Supplemental Table 9. One-way ANOVA with Tukey's multiple comparisons test of *S. epidermidis* 24h on cobalt-chromium

Tukey's multiple comparisons test	Mean Diff.	95% CI of diff.	Below threshold?	Summary	Adj. P value
Untreated vs. Irrigation	0,1848	-0.7533 to 1.123	No	ns	0,9193
Untreated vs. Sonication	1,585	0.6467 to 2.523	Yes	**	0,0028
Untreated vs. RF	1,585	0.6470 to 2.523	Yes	**	0,0028

Irrigation vs. Sonication	1,4	0.4620 to 2.338	Yes	**	0,0061
Irrigation vs. RF	1,4	0.4622 to 2.338	Yes	**	0,0061
Sonication vs. RF	0,0002667	-0.9378 to 0.9383	No	ns	>0.9999

Supplemental Table 10. One-way ANOVA with Tukey's multiple comparisons test of *S. epidermidis* 24h on stainless steel

Tukey's multiple comparisons test	Mean Diff.	95% CI of diff.	Below threshold?	Summary	Adj. P value
Untreated vs. Irrigation	0,002696	-0.01846 to 0.02385	No	ns	0,9755
Untreated vs. Sonication	0,01813	-0.003032 to 0.03928	No	ns	0,0956
Untreated vs. RF	0,02206	0.0009058 to 0.04322	Yes	*	0,0412
Irrigation vs. Sonication	0,01543	-0.005728 to 0.03659	No	ns	0,1687
Irrigation vs. RF	0,01937	-0.001790 to 0.04053	No	ns	0,0733
Sonication vs. RF	0,003938	-0.01722 to 0.02510	No	ns	0,9305

Supplemental Table 11. Kruskal-Wallis with Dunn's multiple comparisons test with Bonferroni correction of *S. epidermidis* 24h on titaniur

Dunn's multiple comparisons test	Dunn's compa	Significant?	Summary	Adj. P value
Untreated vs. Irrigation	-1,498915	No	ns	0,4017
Untreated vs. Sonication	-4,026936	Yes	****	0,0002
Untreated vs. RF	-4,720464	Yes	****	0
Irrigation vs. Sonication	-2,528021	Yes	*	0,0344
Irrigation vs. RF	-3,221549	Yes	***	0,0038
Sonication vs. RF	-0,693527	No	ns	>0.9999

Supplemental Table 12. Kruskal-Wallis with Dunn's multiple comparisons test with Bonferroni correction of *S. epidermidis* 24h on cobalt-

Dunn's multiple comparisons test	Dunn's compa	Significant?	Summary	Adj. P value
Untreated vs. Irrigation	-1,364683	No	ns	0,5171
Untreated vs. Sonication	-4,295398	Yes	****	0,0001
Untreated vs. RF	-4,31777	Yes	****	0
Irrigation vs. Sonication	-2,930714	Yes	*	0,0101
Irrigation vs. RF	-2,953086	Yes	**	0,0094
Sonication vs. RF	-0,022371	No	ns	>0.9999

Supplemental Table 13. Kruskal-Wallis with Dunn's multiple comparisons test with Bonferroni correction of *S. epidermidis* 24h on stainle:

Dunn's multiple comparisons test	Dunn's compa	Significant?	Summary	Adj. P value
Untreated vs. Irrigation	-0,536924	No	ns	>0.9999
Untreated vs. Sonication	-3,24392	Yes	***	0,0035

Untreated vs. RF	-3,780845	Yes	****	0,0005
Irrigation vs. Sonication	-2,706996	Yes	*	0,0204
Irrigation vs. RF	-3,24392	Yes	***	0,0035
Sonication vs. RF	-0,536924	No	ns	>0.9999

Supplemental Table 14. One-way ANOVA with Tukey's multiple comparisons test of *P. aeruginosa* 24h on titanium

Tukey's multiple comparisons test	Mean Diff.	95% CI of diff.	Below threshold?	Summary	Adj. P value
Untreated vs. Irrigation	0,02099	0.007086 to 0.03490	Yes	**	0,0057
Untreated vs. Sonication	0,02405	0.01014 to 0.03795	Yes	**	0,0024
Untreated vs. RF	0,02319	0.009286 to 0.03710	Yes	**	0,0031
Irrigation vs. Sonication	0,003055	-0.01085 to 0.01696	No	ns	0,8929
Irrigation vs. RF	0,0022	-0.01171 to 0.01611	No	ns	0,9552
Sonication vs. RF	-0,000855	-0.01476 to 0.01305	No	ns	0,9971

Supplemental Table 15. One-way ANOVA with Tukey's multiple comparisons test of *P. aeruginosa* 24h on cobalt-chromium

Tukey's multiple comparisons test	Mean Diff.	95% CI of diff.	Below threshold?	Summary	Adj. P value
Untreated vs. Irrigation	0,01417	0.006062 to 0.02228	Yes	**	0,0023
Untreated vs. Sonication	0,01208	0.003971 to 0.02019	Yes	**	0,0061
Untreated vs. RF	0,01531	0.007201 to 0.02342	Yes	**	0,0014
Irrigation vs. Sonication	-0,002091	-0.01020 to 0.006018	No	ns	0,841
Irrigation vs. RF	0,001139	-0.006969 to 0.009247	No	ns	0,9678
Sonication vs. RF	0,00323	-0.004879 to 0.01134	No	ns	0,6013

Supplemental Table 16. One-way ANOVA with Tukey's multiple comparisons test of *P. aeruginosa* 24h on stainless steel

Tukey's multiple comparisons test	Mean Diff.	95% CI of diff.	Below threshold?	Summary	Adj. P value
Untreated vs. Irrigation	0,01779	-0.001555 to 0.03713	No	ns	0,0719
Untreated vs. Sonication	0,01785	-0.001498 to 0.03719	No	ns	0,0709
Untreated vs. RF	0,01898	-0.0003630 to 0.03833	No	ns	0,0544
Irrigation vs. Sonication	0,00005724	-0.01929 to 0.01940	No	ns	>0.9999
Irrigation vs. RF	0,001192	-0.01815 to 0.02054	No	ns	0,997
Sonication vs. RF	0,001135	-0.01821 to 0.02048	No	ns	0,9974

Supplemental Table 17. Kruskal-Wallis with Dunn's multiple comparisons test with Bonferroni correction of *P. aeruginosa* 24h on titaniur

Dunn's multiple comparisons test	Dunn's compa	Significant?	Summary	Adj. P value
Untreated vs. Irrigation	-2,617508	Yes	*	0,0266

Untreated vs. Sonication	-4,496745	Yes	****	0
Untreated vs. RF	-3,668986	Yes	****	0,0007
Irrigation vs. Sonication	-1,879236	No	ns	0,1806
Irrigation vs. RF	-1,051477	No	ns	0,8791
Sonication vs. RF	-0,827759	No	ns	>0.9999

Supplemental Table 18. Kruskal-Wallis with Dunn's multiple comparisons test with Bonferroni correction of *P. aeruginosa* 24h on cobalt-c

Dunn's multiple comparisons test	Dunn's compa	Significant?	Summary	Adj. P value
Untreated vs. Irrigation	-3,624242	Yes	****	0,0009
Untreated vs. Sonication	-2,460905	Yes	*	0,0416
Untreated vs. RF	-4,608604	Yes	****	0
Irrigation vs. Sonication	-1,163337	No	ns	0,7341
Irrigation vs. RF	-0,984362	No	ns	0,9748
Sonication vs. RF	-2,147699	No	ns	0,0952

Supplemental Table 19. Kruskal-Wallis with Dunn's multiple comparisons test with Bonferroni correction of *P. aeruginosa* 24h on stainles

Dunn's multiple comparisons test	Dunn's compa	Significant?	Summary	Adj. P value
Untreated vs. Irrigation	-2,584117	Yes	*	0,0293
Untreated vs. Sonication	-2,60649	Yes	*	0,0274
Untreated vs. RF	-3,355996	Yes	***	0,0024
Irrigation vs. Sonication	-0,022373	No	ns	>0.9999
Irrigation vs. RF	-0,771879	No	ns	>0.9999
Sonication vs. RF	-0,749505	No	ns	>0.9999

Supplemental Table 20. One-way ANOVA with Tukey's multiple comparisons test of *E. coli* 24h on titanium

Tukey's multiple comparisons test	Mean Diff.	95% CI of diff.	Below threshold?	Summary	Adj. P value
Untreated vs. Irrigation	0,003171	0.0008279 to 0.005515	Yes	*	0,0107
Untreated vs. Sonication	0,006704	0.004360 to 0.009047	Yes	****	<0.0001
Untreated vs. RF	0,006646	0.004303 to 0.008990	Yes	****	<0.0001
Irrigation vs. Sonication	0,003532	0.001189 to 0.005876	Yes	**	0,0057
Irrigation vs. RF	0,003475	0.001131 to 0.005818	Yes	**	0,0063
Sonication vs. RF	-5,747E-05	-0.002401 to 0.002286	No	ns	0,9998

Supplemental Table 21. One-way ANOVA with Tukey's multiple comparisons test of *E. coli* 24h on cobalt-chromium

Tukey's multiple comparisons test	Mean Diff.	95% CI of diff.	Below threshold?	Summary	Adj. P value

Untreated vs. Irrigation	0,006731	0.003551 to 0.009912	Yes	***	0,0006
Untreated vs. Sonication	0,007138	0.003957 to 0.01032	Yes	***	0,0004
Untreated vs. RF	0,007661	0.004480 to 0.01084	Yes	***	0,0003
Irrigation vs. Sonication	0,0004065	-0.002774 to 0.003587	No	ns	0,9753
Irrigation vs. RF	0,0009294	-0.002251 to 0.004110	No	ns	0,7874
Sonication vs. RF	0,000523	-0.002658 to 0.003704	No	ns	0,9502

Supplemental Table 22. One-way ANOVA with Tukey's multiple comparisons test of *E. coli* 24h on stainless steel

Tukey's multiple comparisons test	Mean Diff.	95% CI of diff.	Below threshold?	Summary	Adj. P value
Untreated vs. Irrigation	0,006321	0.0008401 to 0.01180	Yes	*	0,0253
Untreated vs. Sonication	0,008243	0.002762 to 0.01372	Yes	**	0,0058
Untreated vs. RF	0,009147	0.003666 to 0.01463	Yes	**	0,0031
Irrigation vs. Sonication	0,001922	-0.003559 to 0.007403	No	ns	0,6867
Irrigation vs. RF	0,002826	-0.002656 to 0.008307	No	ns	0,4057
Sonication vs. RF	0,0009035	-0.004578 to 0.006385	No	ns	0,9499

Supplemental Table 23. Kruskal-Wallis with Dunn's multiple comparisons test with Bonferroni correction of *E. coli* 24h on titanium

Dunn's multiple comparisons test	Dunn's compa	Significant?	Summary	Adj. P value
Untreated vs. Irrigation	-1,610774	No	ns	0,3217
Untreated vs. Sonication	-4,116423	Yes	****	0,0001
Untreated vs. RF	-4,07168	Yes	****	0,0001
Irrigation vs. Sonication	-2,505649	Yes	*	0,0367
Irrigation vs. RF	-2,460905	Yes	*	0,0416
Sonication vs. RF	0,044743	No	ns	>0.9999

Supplemental Table 24. Kruskal-Wallis with Dunn's multiple comparisons test with Bonferroni correction of *E. coli* 24h on cobalt-chromiu

Dunn's multiple comparisons test	Dunn's compa	Significant?	Summary	Adj. P value
Untreated vs. Irrigation	-2,908342	Yes	*	0,0109
Untreated vs. Sonication	-3,109689	Yes	**	0,0056
Untreated vs. RF	-4,854695	Yes	****	0
Irrigation vs. Sonication	-0,201346	No	ns	>0.9999
Irrigation vs. RF	-1,946352	No	ns	0,1548
Sonication vs. RF	-1,745005	No	ns	0,243

Supplemental Table 25. Kruskal-Wallis with Dunn's multiple comparisons test with Bonferroni correction of *E. coli* 24h on stainless steel

Dunn's multiple comparisons test	Dunn's compa	Significant?	Summary	Adj. P value
Untreated vs. Irrigation	-2,326674	No	ns	0,0599
Untreated vs. Sonication	-3,154433	Yes	***	0,0048
Untreated vs. RF	-4,765207	Yes	****	0
Irrigation vs. Sonication	-0,827759	No	ns	>0.9999
Irrigation vs. RF	-2,438533	Yes	*	0,0442
Sonication vs. RF	-1,610774	No	ns	0,3217

Supplemental Table 26. Mixed effect linear model of normalized OD-550 (biofilm) of *S. aureus* 24h across all conditions

Predictors	Estimates	CI	p	djusted (Bonferroni)
(Intercept)	0,03	0.02 – 0.03	**1,64E-10**	**1,97E-09**
Metal [Cobalt-Chromium]	-0,01	-0.01 – 0.00	0,137	1
Metal [Stainless-steel]	0	-0.01 – 0.00	0,198	1
Treatment [Irrigation]	-0,02	-0.03 – -0.01	**4,13E-06**	**4,95E-05**
Treatment [Sonication]	-0,02	-0.03 – -0.02	**1,03E-06**	**1,24E-05**
Treatment [Radiofrequency]	-0,02	-0.03 – -0.02	**4,12E-07**	**4,65E-06**
Metal [Cobalt-Chromium] × Treatment [Irrigation]	0	-0.01 – 0.01	0,828	1,00E+00
Metal [Stainless-steel] × Treatment [Irrigation]	0	-0.01 – 0.01	0,834	1
Metal [Cobalt-Chromium] × Treatment [Sonication]	0	-0.01 – 0.01	0,601	1
Metal [Stainless-steel] × Treatment [Sonication]	0	-0.01 – 0.01	0,676	1
Metal [Cobalt-Chromium] × Treatment [Radiofrequency]	0	-0.01 – 0.01	0,448	1
Metal [Stainless-steel] × Treatment [Radiofrequency]	0	-0.01 – 0.01	0,523	1
Random Effects				
σ^2	0			
$\tau_{00\ Replicate}$	0			
ICC	0,7			
$N_{Replicate}$	36			
Observations	108			
Marginal R^2 / Conditional R^2	0.790 / 0.937			

Correlation of Fixed Effects:

	(Intr)	Metal [cobalt-chromium]	Metal [stainless ste	Treatment [Irrig	Treatment [S
Metal [cobalt-chromium]	-0,707				
Metal [stainless steel]	-0,707	0,5			
Treatment [Irrigation]	-0,707	0,5	0,5		
Treatment [Sonication]	-0,707	0,5	0,5	0,5	
Treatment [Radiofrequency]	-0,707	0,5	0,5	0,5	0,5
Metal [C-C]:Treatment [I]	0,5	-0,707	-0,354	-0,707	-0,354
Metal [SS]:Treatment [I]	0,5	-0,354	-0,707	-0,707	-0,354
Metal[C-C]:Treatment[Sonication]	0,5	-0,707	-0,354	-0,354	-0,707
Metal[SS]:Treatment[Sonication]	0,5	-0,354	-0,707	-0,354	-0,707
tal[CC]:Treatment[Radiofrequency]	0,5	-0,707	-0,354	-0,354	-0,354
etal[SS]:Treatment[Radiofrequency]	0,5	-0,354	-0,707	-0,354	-0,354

Supplemental Table 27. Mixed effect linear model of normalized OD-550 (biofilm) of *S. epidermidis* 24h across all conditions

Predictors	Estimates	CI	p	djusted (Bonferroni)
(Intercept)	0,04	0.03 – 0.04	**3,54E-11**	**4,25E-10**
Metal [Cobalt-Chromium]	-0,01	-0.02 – -0.00	**0,00643**	0,0772
Metal [Stainless-steel]	0	-0.01 – 0.01	0,567	1
Treatment [Irrigation]	-0,01	-0.01 – 0.00	0,286	1
Treatment [Sonication]	-0,03	-0.03 – -0.02	**1,21E-05**	**1,45E-04**
Treatment [Radiofrequency]	-0,03	-0.04 – -0.02	**1,84E-06**	**2,21E-05**
Metal [Cobalt-Chromium] × Treatment [Irrigation]	0	-0.01 – 0.02	0,613	1
Metal [Stainless-steel] × Treatment [Irrigation]	0	-0.01 – 0.02	0,722	1
Metal [Cobalt-Chromium] × Treatment [Sonication]	0,01	-0.00 – 0.02	0,108	1
Metal [Stainless-steel] × Treatment [Sonication]	0,01	-0.01 – 0.02	0,271	1
Metal [Cobalt-Chromium] × Treatment [Radiofrequency]	0,01	0.00 – 0.03	**0,0373**	0,448
Metal [Stainless-steel] × Treatment [Radiofrequency]	0,01	-0.01 – 0.02	0,298	1

Random Effects	
σ^2	0
$\tau_{00\ Replicate}$	0
ICC	0,67
$N_{Replicate}$	36
Observations	108
Marginal R^2 / Conditional R^2	0.729 / 0.910

Correlation of Fixed Effects:

	(Intr)	Metal [cobalt-chromium]	Metal [stainless ste	Treatment [Irrig	Treatment [S
Metal [cobalt-chromium]	-0,707				
Metal [stainless steel]	-0,707	0,5			
Treatment [Irrigation]	-0,707	0,5	0,5		
Treatment [Sonication]	-0,707	0,5	0,5	0,5	
Treatment [Radiofrequency]	-0,707	0,5	0,5	0,5	0,5
Metal [C-C]:Treatment [I]	0,5	-0,707	-0,354	-0,707	-0,354
Metal [SS]:Treatment [I]	0,5	-0,354	-0,707	-0,707	-0,354
Metal[C-C]:Treatment[Sonication]	0,5	-0,707	-0,354	-0,354	-0,707
Metal[SS]:Treatment[Sonication]	0,5	-0,354	-0,707	-0,354	-0,707
tal[CC]:Treatment[Radiofrequency]	0,5	-0,707	-0,354	-0,354	-0,354
tal[SS]:Treatment[Radiofrequency]	0,5	-0,354	-0,707	-0,354	-0,354

Supplemental Table 28. Mixed effect linear model of normalized OD-550 (biofilm) of *P. aeruginosa* 24h across all conditions

Predictors	Estimates	CI	p	-adj. (Bonferroni)
(Intercept)	0,03	0.02 – 0.04	**2,61E-09**	**3,13E-08**
Metal [Cobalt-Chromium]	-0,01	-0.02 – -0.00	**0,0322**	0,386
Metal [Stainless-steel]	0	-0.01 – 0.01	0,463	1
Treatment [Irrigation]	-0,02	-0.03 – -0.01	**0,000107**	**0,00129**
Treatment [Sonication]	-0,02	-0.03 – -0.02	**1,95E-05**	**2,34E-04**
Treatment [Radiofrequency]	-0,02	-0.03 – -0.01	**3,14E-05**	**3,76E-04**
Metal [Cobalt-Chromium] × Treatment [Irrigation]	0,01	-0.01 – 0.02	0,298	1,00E+00
Metal [Stainless-steel] × Treatment [Irrigation]	0	-0.01 – 0.02	0,622	1
Metal [Cobalt-Chromium] ×				

Treatment [Sonication]	0,01	-0.00 – 0.02	0,0744	0,893
Metal [Stainless-steel] × Treatment [Sonication]	0,01	-0.01 – 0.02	0,343	1
Metal [Cobalt-Chromium] × Treatment [Radiofrequency]	0,01	-0.00 – 0.02	0,231	1
Metal [Stainless-steel] × Treatment [Radiofrequency]	0	-0.01 – 0.02	0,518	1
<u>Random Effects</u>				
σ^2	0			
$\tau_{00\ Replicate}$	0			
ICC	0,83			
$N_{Replicate}$	36			
Observations	108			
Marginal R^2 / Conditional R^2	0.666 / 0.943			

<u>Correlation of Fixed Effects:</u>

	(Intr)	Metal [cobalt-chromium]	Metal [stainless ste	Treatment [Irrig	Treatment [S
Metal [cobalt-chromium]	-0,707				
Metal [stainless steel]	-0,707	0,5			
Treatment [Irrigation]	-0,707	0,5	0,5		
Treatment [Sonication]	-0,707	0,5	0,5	0,5	
Treatment [Radiofrequency]	-0,707	0,5	0,5	0,5	0,5
Metal [C-C]:Treatment [I]	0,5	-0,707	-0,354	-0,707	-0,354
Metal [SS]:Treatment [I]	0,5	-0,354	-0,707	-0,707	-0,354
Metal[C-C]:Treatment[Sonication]	0,5	-0,707	-0,354	-0,354	-0,707
Metal[SS]:Treatment[Sonication]	0,5	-0,354	-0,707	-0,354	-0,707
tal[CC]:Treatment[Radiofrequency]	0,5	-0,707	-0,354	-0,354	-0,354
etal[SS]:Treatment[Radiofrequency]	0,5	-0,354	-0,707	-0,354	-0,354

Supplemental Table 29. Mixed effect linear model of normalized OD-550 (biofilm) of *E. coli* 24h across all conditions

<u>Predictors</u>	<u>Estimates</u>	<u>CI</u>	<u>p</u>	<u>djusted (Bonferroni)</u>
(Intercept)	0,01	0.01 – 0.01	**9,38E-11**	**1,13E-09**
Metal [Cobalt-Chromium]	0	-0.00 – 0.00	0,848	1
Metal [Stainless-steel]	0	-0.00 – 0.00	0,069	0,828
Treatment [Irrigation]	0	-0.01 – -0.00	**0,0156**	0,187

Treatment [Sonication]	-0,01	-0.01 – -0.00	**1,17E-05**	**1,40E-04**
Treatment [Radiofrequency]	-0,01	-0.01 – -0.00	**1,32E-05**	**1,58E-04**
Metal [Cobalt-Chromium] × Treatment [Irrigation]	0	-0.01 – -0.00	**0,0497**	5,97E-01
Metal [Stainless-steel] × Treatment [Irrigation]	0	-0.01 – 0.00	0,0799	0,959
Metal [Cobalt-Chromium] × Treatment [Sonication]	0	-0.00 – 0.00	0,803	1
Metal [Stainless-steel] × Treatment [Sonication]	0	-0.00 – 0.00	0,3803	1
Metal [Cobalt-Chromium] × Treatment [Radiofrequency]	0	-0.00 – 0.00	0,561	1
Metal [Stainless-steel] × Treatment [Radiofrequency]	0	-0.01 – 0.00	0,159	1
<u>Random Effects</u>				
σ^2	0			
τ_{00} Replicate	0			
ICC	0,3			
$N_{Replicate}$	36			
Observations	108			
Marginal R^2 / Conditional R^2	0.724 / 0.807			

<u>Correlation of Fixed Effects:</u>

	(Intr)	Metal [cobalt-chromium]	Metal [stainless ste	Treatment [Irrig	Treatment [S
Metal [cobalt-chromium]	-0,707				
Metal [stainless steel]	-0,707	0,5			
Treatment [Irrigation]	-0,707	0,5	0,5		
Treatment [Sonication]	-0,707	0,5	0,5	0,5	
Treatment [Radiofrequency]	-0,707	0,5	0,5	0,5	0,5
Metal [C-C]:Treatment [I]	0,5	-0,707	-0,354	-0,707	-0,354
Metal [SS]:Treatment [I]	0,5	-0,354	-0,707	-0,707	-0,354
Metal[C-C]:Treatment[Sonication]	0,5	-0,707	-0,354	-0,354	-0,707
Metal[SS]:Treatment[Sonication]	0,5	-0,354	-0,707	-0,354	-0,707
tal[CC]:Treatment[Radiofrequency]	0,5	-0,707	-0,354	-0,354	-0,354
tal[SS]:Treatment[Radiofrequency]	0,5	-0,354	-0,707	-0,354	-0,354

Supplemental Table 30. One-way ANOVA with Tukey's multiple comparisons test of *S. aureus* robust biofilm on titanium

Tukey's multiple comparisons test	Mean Diff.	95% CI of diff.	Below threshold?	Summary	Adj. P value
Untreated vs. Irrigation	0,01195	-0.003242 to 0.02715	No	ns	0,1309
Untreated vs. Sonication	0,01781	0.002618 to 0.03301	Yes	*	0,0232
Untreated vs. RF	0,01944	0.004241 to 0.03463	Yes	*	0,0146
Irrigation vs. Sonication	0,00586	-0.009334 to 0.02105	No	ns	0,624
Irrigation vs. RF	0,007483	-0.007711 to 0.02268	No	ns	0,4411
Sonication vs. RF	0,001623	-0.01357 to 0.01682	No	ns	0,9852

Supplemental Table 31. One-way ANOVA with Tukey's multiple comparisons test of *S. aureus* robust biofilm on cobalt-chromium

Tukey's multiple comparisons test	Mean Diff.	95% CI of diff.	Below threshold?	Summary	Adj. P value
Untreated vs. Irrigation	0,01189	0.005239 to 0.01854	Yes	**	0,002
Untreated vs. Sonication	0,01489	0.008239 to 0.02154	Yes	***	0,0004
Untreated vs. RF	0,01567	0.009021 to 0.02232	Yes	***	0,0003
Irrigation vs. Sonication	0,003	-0.003650 to 0.009650	No	ns	0,5089
Irrigation vs. RF	0,003782	-0.002867 to 0.01043	No	ns	0,3309
Sonication vs. RF	0,0007821	-0.005868 to 0.007432	No	ns	0,9805

Supplemental Table 32. One-way ANOVA with Tukey's multiple comparisons test of *S. aureus* robust biofilm on stainless steel

Tukey's multiple comparisons test	Mean Diff.	95% CI of diff.	Below threshold?	Summary	Adj. P value
Untreated vs. Irrigation	0,0186	0.007832 to 0.02938	Yes	**	0,0025
Untreated vs. Sonication	0,02305	0.01228 to 0.03382	Yes	***	0,0006
Untreated vs. RF	0,02581	0.01504 to 0.03658	Yes	***	0,0003
Irrigation vs. Sonication	0,004446	-0.006327 to 0.01522	No	ns	0,5757
Irrigation vs. RF	0,007207	-0.003566 to 0.01798	No	ns	0,219
Sonication vs. RF	0,002761	-0.008012 to 0.01353	No	ns	0,8433

Supplemental Table 33. Kruskal-Wallis with Dunn's multiple comparisons test with Bonferroni correction of *S. aureus* robust biofilm on ti

Dunn's multiple comparisons test	Dunn's compa	Significant?	Summary	Adj. P value
Untreated vs. Irrigation	-1,812121	No	ns	0,2099
Untreated vs. Sonication	-4,026936	Yes	****	0,0002
Untreated vs. RF	-4,407258	Yes	****	0
Irrigation vs. Sonication	-2,214814	No	ns	0,0803
Irrigation vs. RF	-2,595136	Yes	*	0,0284

Sonication vs. RF	-0,380321	No	ns	>0.9999

Supplemental Table 34. Kruskal-Wallis with Dunn's multiple comparisons test with Bonferroni correction of *S. aureus* robust biofilm on c

Dunn's multiple comparisons test	Dunn's compa	Significant?	Summary	Adj. P value
Untreated vs. Irrigation	-2,080583	No	ns	0,1124
Untreated vs. Sonication	-4,07168	Yes	****	0,0001
Untreated vs. RF	-4,720464	Yes	****	0
Irrigation vs. Sonication	-1,991096	No	ns	0,1394
Irrigation vs. RF	-2,63988	Yes	*	0,0249
Sonication vs. RF	-0,648784	No	ns	>0.9999

Supplemental Table 35. Kruskal-Wallis with Dunn's multiple comparisons test with Bonferroni correction of *S. aureus* robust biofilm on s

Dunn's multiple comparisons test	Dunn's compa	Significant?	Summary	Adj. P value
Untreated vs. Irrigation	-1,879236	No	ns	0,1806
Untreated vs. Sonication	-3,691358	Yes	****	0,0007
Untreated vs. RF	-5,212545	Yes	****	0
Irrigation vs. Sonication	-1,812121	No	ns	0,2099
Irrigation vs. RF	-3,333408	Yes	***	0,0026
Sonication vs. RF	-1,521287	No	ns	0,3846

mium

teel

n

chromium

ss steel

ו

:hromium

;s steel

ım

Treatment [	Metal [CC	Metal [SS	Metal[C	Metal[SS	Metal[CC]:Treatment[Radiofrequency]
-0,354					
-0,354	0,5				
-0,354	0,5	0,25			
-0,354	0,25	0,5	0,5		
-0,707	0,5	0,25	0,5	0,25	
-0,707	0,25	0,5	0,25	0,5	0,5

Treatment [	Metal [CC	Metal [SS	Metal[C	Metal[SS	Metal[CC]:Treatment[Radiofrequency]
-0,354					
-0,354	0,5				
-0,354	0,5	0,25			
-0,354	0,25	0,5	0,5		
-0,707	0,5	0,25	0,5	0,25	
-0,707	0,25	0,5	0,25	0,5	0,5

Treatment [	Metal [CC	Metal [SS	Metal[C	Metal[SS	Metal[CC]:Treatment[Radiofrequency]
-0,354					
-0,354	0,5				
-0,354	0,5	0,25			
-0,354	0,25	0,5	0,5		
-0,707	0,5	0,25	0,5	0,25	
-0,707	0,25	0,5	0,25	0,5	0,5

Treatment [	Metal [CC	Metal [SS	Metal[C	Metal[SS	Metal[CC]:Treatment[Radiofrequency]
-0,354					
-0,354	0,5				
-0,354	0,5	0,25			
-0,354	0,25	0,5	0,5		
-0,707	0,5	0,25	0,5	0,25	
-0,707	0,25	0,5	0,25	0,5	0,5

tanium

obalt-chromium

tainless steel
